# HIST1H2BJ is associated with poor prognosis and an immune-altered tumor microenvironment in glioma

**DOI:** 10.3389/fimmu.2026.1929885

**Published:** 2026-09-16

**Authors:** Lupeng Zhang, Yue Li, Chen Shi, Yong Zhang, Ruijuan Liu, Chiwen Qu, Sihua Peng, Xiaoning Peng

**Affiliations:** 1 College of Mathematics and Statistics, Hunan Normal University, Changsha, Hunan, China; 2 Department of Biochemistry and Molecular Biology, Jishou University School of Medicine, Jishou, Hunan, China; 3 Department of Pathology, Xiangtan Central Hospital, Xiangtan, Hunan, China; 4 Department of Pathology and Pathophysiology, Hunan Normal University School of Medicine, Changsha, Hunan, China; 5 Department of Oncology, Weifang Hospital of Traditional Chinese Medicine, Weifang, China; 6 School of Public Health and Management, Youjiang Medical University for Nationalities, Baise, Guangxi, China; 7 Department of Epidemiology and Biostatistics, College of Public Health, University of Georgia, Athens, GA, United States

**Keywords:** biomarker, glioma, HIST1H2BJ, prognosis prediction, tumor microenvironment

## Abstract

**Background:**

Glioma, the most prevalent and aggressive primary malignant brain tumor, is associated with poor clinical outcomes. There is an urgent need for novel biomarkers to predict survival and therapeutic response, as existing markers offer limited prognostic utility.

**Methods:**

Single-cell RNA sequencing data from glioma patients were analyzed to identify CD8+ T cell-related genes. Four machine learning algorithms (XGBoost, Gradient Boosting Machine, LASSO, and Random Forest) were employed to screen genes associated with glioma prognosis. The expression of *HIST1H2BJ* was detected in clinical samples. Flow cytometry was performed to assess cell apoptosis, while Transwell assays were conducted to evaluate cell invasion and migration capabilities, respectively. A novel prognostic model, the Immune and Metabolic Prognostic Model of Glioma (IMPMG), was developed, and its accuracy was evaluated using receiver operating characteristic (ROC) curves and decision curve analysis (DCA). Additionally, potential small-molecule compounds were screened via molecular docking, and the binding stability was evaluated by molecular dynamics (MD) simulations.

**Results:**

*HIST1H2BJ* was identified as a critical gene associated with glioma prognosis. Functional assays confirmed that HIST1H2BJ knockdown promoted cell apoptosis and inhibited cell invasion and migration. Glioma patients with high *HIST1H2BJ* expression were closely associated with an immunosuppressive tumor microenvironment, with significantly increased infiltration of M2 macrophages and regulatory T cells (Tregs), as well as upregulated immune checkpoint expression. The IMPMG model, integrating *HIST1H2BJ*, immune-metabolic genes, and clinical variables, demonstrated superior prognostic accuracy (AUC > 0.86) and clinical utility compared to existing models. Furthermore, molecular docking identified VX-680 and A.770041 as candidate compounds associated with *HIST1H2BJ*-high drug-sensitivity prediction; their direct binding to *HIST1H2BJ* was assessed only in silico by molecular docking and dynamics simulations and requires experimental validation.

**Conclusion:**

This study establishes *HIST1H2BJ* as an independent prognostic factor for glioma patient survival and a candidate prognostic biomarker whose therapeutic relevance requires further functional and preclinical investigation.

## 1 Introduction

Glioma represents the most frequent and histologically heterogeneous primary intracranial tumor, accounting for approximately 70% of all malignant brain tumors (1). Based on the 5th edition of the World Health Organization (WHO) classification of central nervous system tumors, gliomas are stratified into low-grade gliomas (LGG) and high-grade gliomas, including glioblastoma (GBM) (2, 3). These neoplasms are marked by profound molecular and cellular heterogeneity, as well as inherent resistance to conventional therapies. The clinical outcome for patients with GBM remains particularly poor, with a median overall survival of approximately 15–18 months even under maximal therapy (4). Although current biomarkers provide some prognostic information, their clinical applicability remains constrained, underscoring the demand for more reliable prognostic indicators and dynamic biomarkers capable of monitoring therapeutic response.

Advancements in glioma research increasingly hinge on deciphering tumor-specific molecular alterations. Comprehensive genomic profiling has elucidated key molecular features, including mutations in isocitrate dehydrogenase (IDH), amplifications of epidermal growth factor receptor (EGFR), and deletions of *CDKN2A/B*, which have subsequently refined glioma classification and prognostic evaluation (5, 6). Such markers are gradually being incorporated into clinical practice and demonstrate substantial predictive utility (7–10). Nonetheless, the limitations of these biomarkers are evident. The prognostic value of IDH mutations and 1p/19q co-deletion remains largely confined to specific molecular subtypes. The recent phase III INDIGO trial demonstrated that the brain-penetrant IDH1/2 inhibitor vorasidenib significantly prolonged progression-free survival in patients with residual or recurrent grade 2 glioma (11, 12); however, for other subtypes or high-grade gliomas, achieving long-term control of tumor progression and managing treatment resistance remain highly challenging (13, 14).While these markers provide a robust foundation for glioma classification, complementary biomarkers are needed to characterize heterogeneity beyond established molecular subtypes and address treatment resistance. Accordingly, identifying novel prognostic indicators and delineating their mechanistic roles in glioma pathogenesis may further refine personalized prognostication and therapeutic development.

Emerging evidence indicates that histone modifications serve as pivotal epigenetic regulators that dynamically reshape the tumor microenvironment (TME) and modulate tumor progression (15). The human genome contains 85 histone genes, comprising 68 classical histone genes and 17 histone variant genes (16). In gliomas, histone H3 lysine methylation and acetylation can specifically upregulate *SOX9* and *FOXG1*, promoting malignant proliferation and migration of gliomas (17). Histone H3 lysine 27 (H3K27M) is a typical oncoprotein that frequently mutates in gliomas and promotes tumor development (18). Although histone modifications such as methylation and acetylation have received significant attention (19), the role of histone family genes in glioma and their relationship with glioma prognosis are still unclear.

The advent of single-cell RNA sequencing (scRNA-seq) has revolutionized the dissection of cellular diversity and interaction networks within tumors (20). Large-scale consortia such as the Chinese Glioma Genome Atlas (CGGA) and The Cancer Genome Atlas (TCGA) have provided invaluable multi-omic resources that facilitate molecular taxonomy and therapeutic discovery. Through integrated analysis of multi-omics data from GEO, CGGA, and TCGA, we identified *HIST1H2BJ*, a gene encoding a histone H2B variant (also known as *H2BC11* in current HGNC nomenclature), as a novel prognostic determinant in glioma. We subsequently confirmed its elevated expression in glioma tissues and cell lines, and functionally characterized its role via knockdown experiments, while further exploring its association with the TME and predicted immunotherapy response. To our knowledge, this study characterizes *HIST1H2BJ* as a candidate regulator linking epigenetic remodeling and anti-tumor immunity in glioma, offering novel mechanistic insights and potential therapeutic avenues.

## 2 Materials and methods

### 2.1 Cell lines, clinical specimens, and ethics statement

Human glioma cell lines (U87, U251, A172, and Hs683) were obtained from GeneChem (Shanghai, China), the human astrocyte cell line HEB was acquired from BeNa Culture Collection (Henan, China). All glioma cells were cultured in a humidified atmosphere containing 5% CO_2_ at 37 °C, according to the suppliers’ recommendations. Glioma specimens (n = 27) and non-tumor brain tissue specimens (n = 5) were collected from patients undergoing surgical resection at Weifang Hospital of Traditional Chinese Medicine. None of the patients had received preoperative radiotherapy or chemotherapy. All specimens were histologically confirmed. The study protocol was approved by the Ethics Committee of Weifang Hospital of Traditional Chinese Medicine, and all experiments were conducted in accordance with relevant guidelines and regulations. Written informed consent was obtained from all participants.

### 2.2 Retrieval and preprocessing of glioma data from public databases

scRNA-seq data, gene expression profiles and clinical information of glioma samples were downloaded from the GEO (GSE182109), CGGA (including CGGA1 and CGGA2 datasets) and TCGA database (6, 21). Additionally, Somatic mutations, CNVs, and DNA methylation data were also from TCGA.

### 2.3 scRNA-seq analysis and cell-cell communication analysis

The single-cell RNA sequencing (scRNA-seq) data for the GSE182109 dataset were obtained from the Gene Expression Omnibus (GEO) database. Subsequent data analysis was performed strictly following the analytical pipeline described by Abdelfattah et al. (22). This dataset comprises 44 tumor samples from 18 patients with primary glioblastoma, and libraries were prepared using the 10x Genomics Chromium 3’ Transcriptomics kit V1 targeting poly(A) RNA. Raw FASTQ files were processed using Cell Ranger v5 with default parameters, aligning reads to the GRCh38 reference genome. To address multi-mapping issues, only uniquely mapped reads were retained for quantification. Since the original study had already performed stringent quality control (including the removal of doublets and RNA contamination), we directly extracted the high-quality cell expression matrices that had passed this quality control.

The Seurat R package (v4.0) was used to construct Seurat objects, and further standard filtering was applied to the included cells: cells with 500–4,000 UMIs, 500–6,000 detected genes, and<10% mitochondrial gene content were retained. After this filtering, a total of 201,241 cells were retained for downstream analysis. Batch effect correction was performed using the Harmony R package with patient ID as the grouping variable. Subsequently, principal component analysis (PCA) was conducted for dimensionality reduction, and the top 30 principal components (PCs) were selected for uniform manifold approximation and projection (UMAP) visualization. Finally, clustering was performed using the Louvain algorithm at a resolution of 0.8.

Intercellular communication networks were inferred from the single-cell data using the CellChat R package. To define the high (*HIST1H2BJ*^high^) and low (*HIST1H2BJ*^low^) expression groups, we calculated the median *HIST1H2BJ* expression in tumor cells for each patient. Patients were then stratified into high- and low-expression groups based on the overall median of these patient-level values. CellChat objects were constructed separately for the *HIST1H2BJ*^high^ and *HIST1H2BJ*^low^. The compareInteractions and netVisual_diffInteraction functions were utilized to descriptively compare the number and strength of inferred ligand–receptor interactions, the signaling pathways enriched in each cell subpopulation, and the communication probabilities of CD8+ T cell–derived ligand–receptor pairs between the two groups.

### 2.4 Identification of prognostic genes for glioma

Through univariate Cox regression analysis, multivariate Cox regression analysis, and ROC curve analysis, genes with prognostic potential were identified in the CGGA1 dataset. Subsequently, four machine learning algorithms (XGBoost, Gradient Boosting Machine, LASSO regression, Random Forest) were employed to identify crucial prognostic genes. Detailed information is listed in the **Supplementary Materials**.

### 2.5 Establishment of *HIST1H2BJ*-knockdown stable cell lines

The U251 and U87 cells were established stable cell lines using lentiviral methods including synthesized *HIST1H2BJ* inhibitor oligonucleotides, following a previously established study protocol (23). The shRNA sequences targeting human H2BC11 (NM_021058; clones 106264-1, 106265-1, and 106266-2) were cloned into the GV493 lentiviral vector (GeneChem, Shanghai, China), with scrambled shRNA (CON313) as the negative control. U87 cells (MEM with 10% FBS) and U251 cells (DMEM with 10% FBS) were infected at an MOI of 5 and 10, respectively, using HiTransG A at 20% confluency, and transduction efficiency was confirmed by GFP expression at 72 h post-infection.

### 2.6 MTT assay, soft agar colony formation assay, and apoptosis assay

For the MTT assay, cells were trypsinized, and single-cell suspensions were prepared. After culturing under the same general conditions, cell viability was calculated after mixing MTT solution (Biotech, Shanghai, China). The following method was employed to determine cell viability: % cell viability = (OD shRNA treated/OD control) × 100.

For the soft agar colony formation assay, cells were planted into 6-well plates and cultivated in an incubator for 2 weeks until colonies appeared. The cell colonies were fastened with 4% paraformaldehyde and dyed by crystal violet. The colonies were then observed, calculated, and photographed.

For the apoptosis assay, single-cell suspensions were mixed with 200 μL of precooled 1× binding buffer solution and 10 μL of Annexin V-APC staining solution in proper order and incubated in the darkroom. The glioma cell apoptosis rates were detected by flow cytometry.

### 2.7 Transwell assay

The transwell assay was utilized to analyze the migration and invasion abilities of glioma after transfection with shRNA-*HIST1H2BJ*, following a previously established study protocol (23).

### 2.8 Analysis of tumor immune microenvironment

The immune score, stromal score, and tumor purity were evaluated by the ESTIMATE algorithm. Additionally, the infiltration abundance of human immune cell subsets in each glioma sample was computed by employing algorithms including MCPcounter, xCell, EPIC, TIMER, quantiseq, and CIBERSORT (24). Furthermore, immune-related feature scores, such as T-cell inflammation marker scores, were computed using the ssGSEA method implemented in the “GSVA” R package (25, 26).

### 2.9 Immunotherapy response prediction

We utilized ImmuCellAI to predict the response of glioma patients to immunotherapy and analyzed the proportion of glioma patients responding to immunotherapy in *HIST1H2BJ* high and *HIST1H2BJ* low groups (27). The TIDE scores obtained from genomic data of gliomas were used to predict the clinical response of gliomas to ICBs (28).

### 2.10 Molecular docking and molecular dynamics simulations

The protein structure was sourced from UniProt, specifically identified as a human species within the database. The complete AlphaFold-predicted structure was chosen as the representative protein structure file. The protein targets were dehydrated and hydrogenated using AutoDock Tools 1.5.6 software, and then subjected to molecular docking.

Molecular dynamics simulations were conducted to investigate the interactions within protein-ligand complexes using GROMACS software version 2019.6. Visualization, analysis, and trajectory animation were accomplished using VMD software version 1.9.3, in conjunction with PyMOL version 2.4.1. Additional bioinformatic analyses were performed as follows. Differential drug sensitivity (half-maximal inhibitory concentration, IC50) between subgroups was predicted from expression profiles using the pRRophetic R package to prioritize candidate compounds for molecular docking. Recurrent somatic copy-number alterations were identified with GISTIC2.0, and differences in mutation and copy-number frequencies between subgroups were summarized with maftools. Overexpression of *HIST1H2BJ* at the mRNA and protein level was further corroborated using the GEPIA and Human Protein Atlas (HPA) databases. The seven-step cancer-immunity cycle was scored with the Tracking Tumor Immunophenotype (TIP) tool. A fixed-effects meta-analysis was used to derive a pooled hazard ratio for *HIST1H2BJ* across the CGGA1, CGGA2, and TCGA cohorts.

### 2.11 IMPMG prognostic model

To enhance the accuracy of predicting glioma patient prognosis, we adopted a two-step approach to construct a prognostic model. The CGGA1 cohort was designated as the training set, while the CGGA2 and TCGA cohorts served as independent external validation sets. Firstly, we conducted a stepwise multivariate Cox regression analysis using immune and metabolic function-related genes significantly correlated with *HIST1H2BJ* to derive an IMGscore (immune and metabolic genes score). Subsequently, *HIST1H2BJ*, IMGscore, and clinical variables were incorporated into a stepwise multivariate Cox regression to develop the Immune and Metabolic Prognostic Model of Glioma (IMPMG). In both steps, each variable was weighted by its Cox regression coefficient, and the risk score was computed as the linear combination of these weighted terms, with the complete set of coefficients given by the equations below. The cutoff values for risk stratification were determined based on the median score of the training set (CGGA1) and were consistently applied to the validation cohorts. The specific steps are outlined as follows:


IMGscore=−ALDH5A1∗0.47−CHGB∗0.17−ALDOC∗0.10+THRA∗0.23−ITPK1∗0.16−AKR1C3∗0.15+GSRA4∗0.16−SEMA6B∗0.12−LCAT∗0.18

IMPMG=HIST1H2BJ∗0.21+PRS_type∗0.71+Grade∗0.55+Age∗0.28−IDH_mutation_status∗0.26−1p19q_codeletion_status∗0.78+IMGscore∗0.20

The incremental prognostic value of the IMPMG model relative to a clinical-variables-only baseline model was quantified using the integrated discrimination improvement (IDI) and net reclassification improvement (NRI), with 95% confidence intervals estimated by bootstrap resampling (1,000 repetitions) using the R package survIDINRI.

### 2.12 Statistical analysis

All statistical analyses were executed through R software (version 4.0) and/or SPSS software (version 26.0). All p-values were two-sided, and statistical significance was defined as P< 0.05. Group comparisons used the Wilcoxon rank-sum test for continuous variables and Fisher’s exact or chi-square tests for categorical variables, while *in vitro* data were analyzed by one-way ANOVA with Tukey’s *post hoc* test. Survival differences were assessed using Kaplan–Meier curves and the log-rank test, and time-dependent ROC analysis was used to evaluate predictive accuracy. Where multiple comparisons were performed, the false discovery rate was controlled using the Benjamini–Hochberg procedure.

## 3 Results

### 3.1 Identification of HIST1H2BJ as a prognostic gene in glioma

We analyzed the scRNA-seq data (GSE182109) in primary adult glioma samples by Seurat (4.3.0). Unsupervised clustering via UMAP revealed eight distinct cell populations, including astrocytes, monocytes, macrophages, T cells, tissue stem cells, endothelial cells, B cells, and smooth muscle cells (**Figure 1A**). Subsequent subclustering of 17,843 T cells identified four subsets: CD8+ T, naïve T, Treg, and CD4+ T cells (**Figure 1B**). To characterize the transcriptional heterogeneity among these T cell subsets, differential expression analysis (logFC > 1, FDR< 0.05) was performed. The results identified *CD8A*, *GZMK*, and *GZMH* as the top three specifically upregulated markers for the CD8+ T cell subset (**Figures 1C, D**). Furthermore, Gene Set Enrichment Analysis (GSEA) revealed distinct functional signatures across the different T cell subpopulations. Specifically, the CD8+ T cell subset was primarily enriched in cytotoxicity-related genes (*CCL4L2*, *GZMH*, *CCL5*, *NKG7*, *CD8A/B*) and associated pathways, including cell killing, cell destruction, body and structural organization, and leukocyte-mediated cytotoxicity (**Figure 1E**).

**Figure 1:**
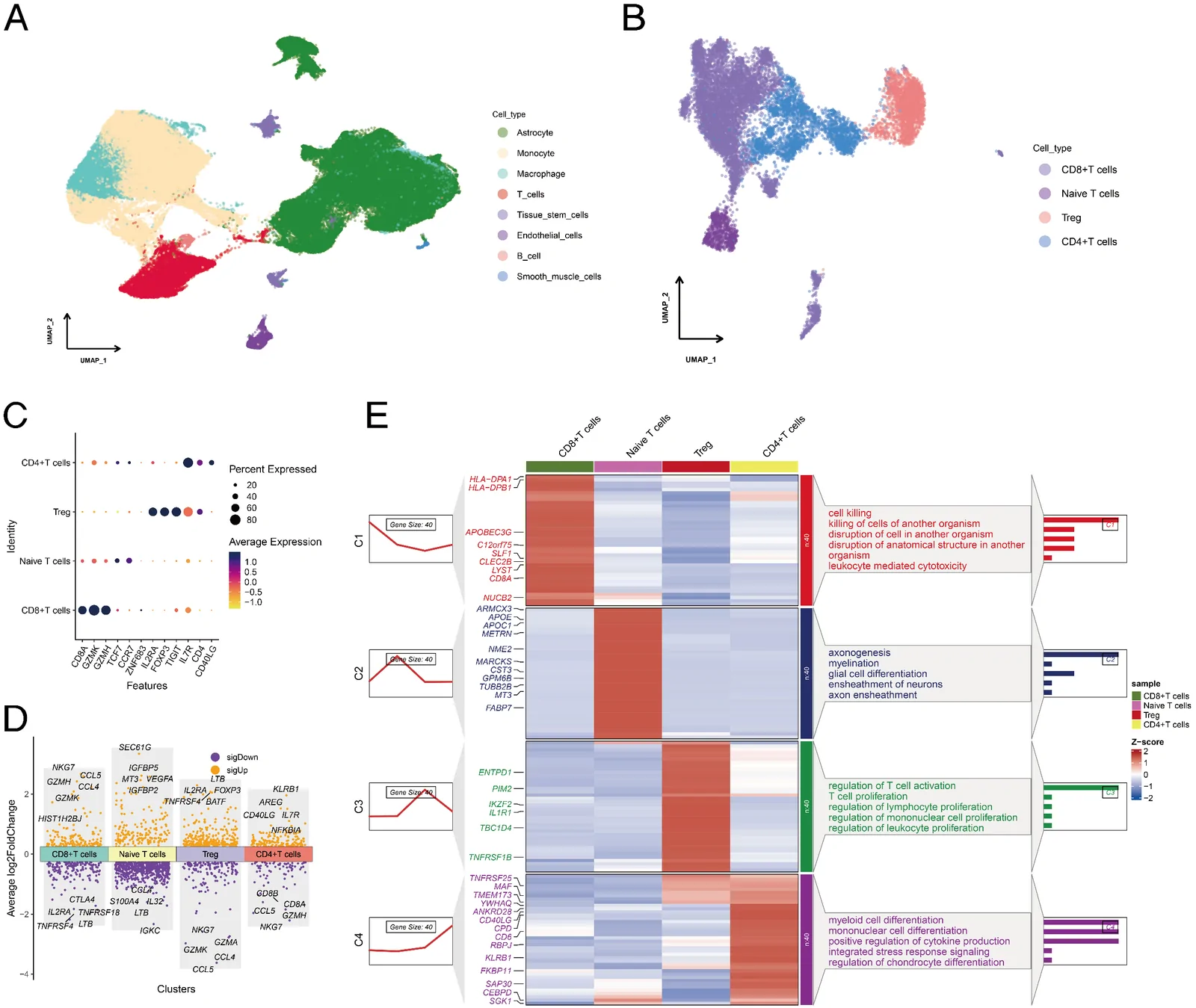
Single-cell RNA-seq profiling of glioma. **(A)** UMAP plot showing the cell subtypes of glioma. **(B)** UMAP plot showing the clusters of T cells. **(C)** Bubble plots showing the markers to each cell population. **(D)** Manhattan plot showing differential expressed genes between T cell clusters. **(E)** Heatmap showing the different T cell populations and their markers and the associated enriched pathways (FDR<0.01).

To completely eliminate information leakage during the feature selection process and to identify robust prognostic biomarkers from the whole transcriptome, we established a rigorous Bootstrap resampling framework (B = 500) in the CGGA1 cohort. In each iteration, a Bootstrap training set was constructed via random sampling with replacement. Within each Bootstrap training set, we first performed Kaplan–Meier survival analysis and univariate Cox regression analysis on 16,381 genes, identifying 4,642 prognosis-associated genes (p< 0.001, occurrence frequency > 70%, **Supplementary Table S1**). Subsequently, these 4,642 genes were incorporated into a multivariate Cox regression model adjusting for prespecified clinical covariates, yielding 1,746 independent prognostic genes (p< 0.001, occurrence frequency > 70%, **Supplementary Table S2**). Further time-dependent receiver operating characteristic (ROC) curve analysis on these 1,746 genes retained 192 genes with excellent predictive performance (AUC > 0.7, occurrence frequency > 70%, **Supplementary Table S3**). Finally, through clinical correlation analysis, 115 genes significantly associated with at least seven clinical characteristics were retained (occurrence frequency > 70%, **Supplementary Table S4**).

To further refine the candidate prognostic genes, we applied four distinct machine-learning algorithms XGBoost, Random Forest, LASSO regression, and Gradient Boosting Machine to the aforementioned Bootstrap-derived candidate gene set. Across the 500 iterations, XGBoost, Random Forest, LASSO, and Gradient Boosting Machine identified 38, 26, 47, and 40 genes, respectively, in over 70% of the iterations, thereby forming their respective core gene sets (**Supplementary Table S5**). Ultimately, we calculated the intersection of these four algorithm-derived core gene sets along with the differentially expressed genes (DEGs) of the CD8^+^ T cell subset. *HIST1H2BJ* was identified as the only gene present across all five sets (**Supplementary Figure S1**). This finding suggests a potential association between *HIST1H2BJ* and CD8^+^ T cell-related transcriptional signatures in glioma. However, since *HIST1H2BJ* is a histone-coding gene, its expression levels may also reflect cellular proliferative activity or intrinsic tumor-cell biological behaviors. Therefore, its predominant cellular source warrants further validation through single-cell technologies.

To validate the prognostic utility of *HIST1H2BJ*, we stratified glioma patients into *HIST1H2BJ*^high^ and *HIST1H2BJ*^low^ based on median expression threshold. KM survival curves were plotted in three independent cohorts (CGGA1, CGGA2, TCGA) and demonstrated that the OS of glioma patients in *HIST1H2BJ*^high^ was shorter than that in *HIST1H2BJ*^low^ (log-rank p< 0.0001, **Figures 2A–C**). The prognostic accuracy of *HIST1H2BJ* was further evidenced by ROC analysis, yielding AUC > 0.65 (**Figures 2D–F**). *HIST1H2BJ* was confirmed as an independent prognostic factor for glioma patients through a multivariate Cox model that adjusted for PRS type, histology, WHO grade, age, gender, chemotherapy and radiotherapy status, IDH status, and 1p/19q co-deletion (p< 0.05, **Figures 2G–I**). Furthermore, a meta-analysis revealed a pooled hazard ratio (HR > 1, p< 0.05), establishing its potential for risk stratification across glioma cohorts, although subtype-stratified validation (for example, within IDH-wildtype glioblastoma) remains to be demonstrated (**Figure 2J**).

**Figure 2:**
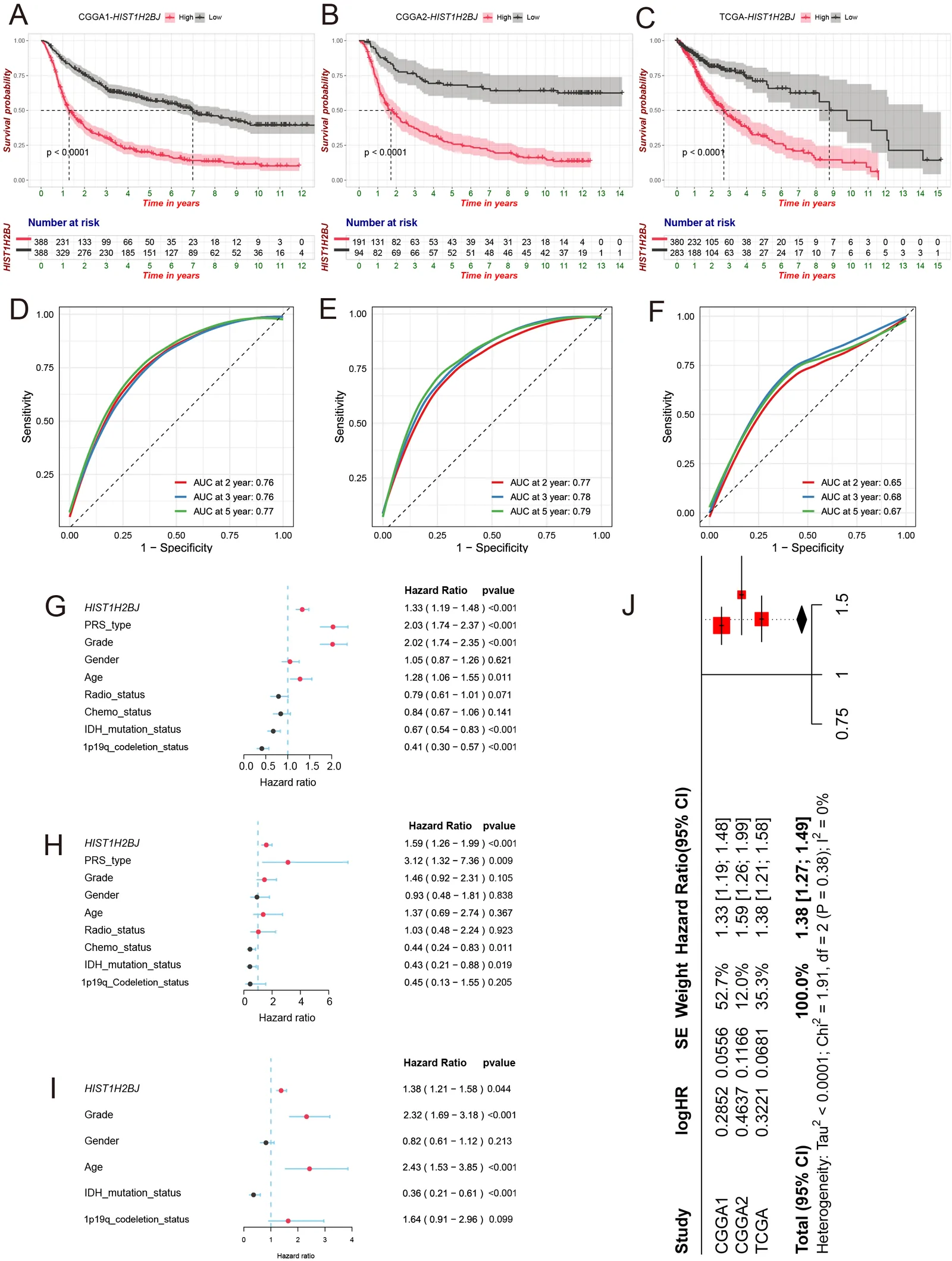
Prognostic analysis of different *HIST1H2BJ* subgroups. **(A–C)** The survival differences between HIST1H2BJhigh and HIST1H2BJlow patients in **(A)** CGGA1, **(B)** CGGA2 and **(C)** TCGA cohorts. **(D–F)** The time-dependent ROC and AUC of *HIST1H2BJ* in **(D)** CGGA1, **(E)** CGGA2 and **(F)** TCGA cohorts, respectively. **(G–I)** Multivariate Cox regression analyses of *HIST1H2BJ* level with PRS_type, histology, grade, age, gender, Chemo_status, Radio_status, IDH_status, and 1p/19q co-deletion_status in the **(G)**CGGA1, **(H)**CGGA2 and **(I)**TCGA cohorts. **(J)** The meta-analysis of prognostic values of *HIST1H2BJ* for patients across three cohorts. A fixed effects model was used to calculate pooled HR value. A fixed-effects model was used due to low heterogeneity (I^2^ = 0%, p = 0.38). The size of the data markers (squares) corresponds to the weight of each study in the pooled analysis. The diamond represents the overall pooled HR and its 95% CI.

### 3.2 *HIST1H2BJ* expression in gliomas

Integrated analysis of the GEPIA and HPA databases revealed pronounced overexpression of HIST1H2BJ in glioma compared to non-tumor tissues (**Supplementary Figures S2A–E**). The expression of *HIST1H2BJ* exhibited notable correlations with critical clinicopathological parameters, including PRS classification, WHO grade, chemotherapy treatment status, IDH mutation status, and the 1p/19q co-deletion (**Supplementary Figures S3A–F**).

The mRNA expression levels of *HIST1H2BJ* in glioma cell lines (A172, Hs683, U87, and U251) were significantly higher than those in normal glial cells (**Supplementary Figure S4A**). Immunohistochemistry (IHC) staining revealed that both the staining intensity and positive expression rate of HIST1H2BJ were markedly stronger in glioma tissues compared to non-tumor brain tissues (**Supplementary Figure S4B**). Additionally, HIST1H2BJ expression was significantly upregulated in a WHO grade-dependent manner; however, no significant correlations with patient age or gender were observed (**Supplementary Table S6**).

To elucidate the mechanism of up-regulated *HIST1H2BJ* expression in glioma, we examined the mutation, CNV, and methylation status of *HIST1H2BJ* using omics data from TCGA. We found that no somatic mutations were detected in *HIST1H2BJ* of glioma patients (**Supplementary Figure S5A**), and the CNV of *HIST1H2BJ* showed no correlation with its expression (**Supplementary Figures S5B, C**). However, DNA methylation analysis revealed significant hypomethylation in glioma samples with high *HIST1H2BJ* expression (**Supplementary Figure S5D**). Furthermore, by analyzing the relationship between CpG site methylation and *HIST1H2BJ* gene expression in glioma, we found that three CpG sites (cg04289040, cg07303012, and cg01380658) within the *HIST1H2BJ* promoter exhibited strong inverse correlations between methylation and expression (**Supplementary Figures S5E-G**). These results suggest that promoter hypomethylation may contribute to *HIST1H2BJ* overexpression in glioma; however, this association is correlative and would require experimental confirmation, for example by bisulfite sequencing or methylation-specific PCR.

### 3.3 The effect of *HIST1H2BJ* on the proliferation, migration, and invasion of glioma cells

To investigate the biological significance of *HIST1H2BJ* in glioma, we established stable *HIST1H2BJ*-knockdown U87 and U251 glioma cells using a lentivirus system (**Supplementary Figure S6**). Successful infection was confirmed by fluorescence microscopy and qRT-PCR (**Supplementary Figure S6**). MTT assays and colony formation assays showed that inhibition of *HIST1H2BJ* expression reduced glioma cell proliferation (**Figures 3A–D**). Transwell migration and Matrigel invasion assays revealed that *HIST1H2BJ* silencing suppressed both migration and invasion of glioma cells (**Figures 3E–H**). Flow cytometry analysis demonstrated that inhibiting *HIST1H2BJ* expression promoted apoptosis of glioma cells (**Figure 3I**). Collectively, these results indicate that *HIST1H2BJ* contributes to glioma progression through coordinated regulation of proliferation, invasion and apoptosis resistance.

**Figure 3:**
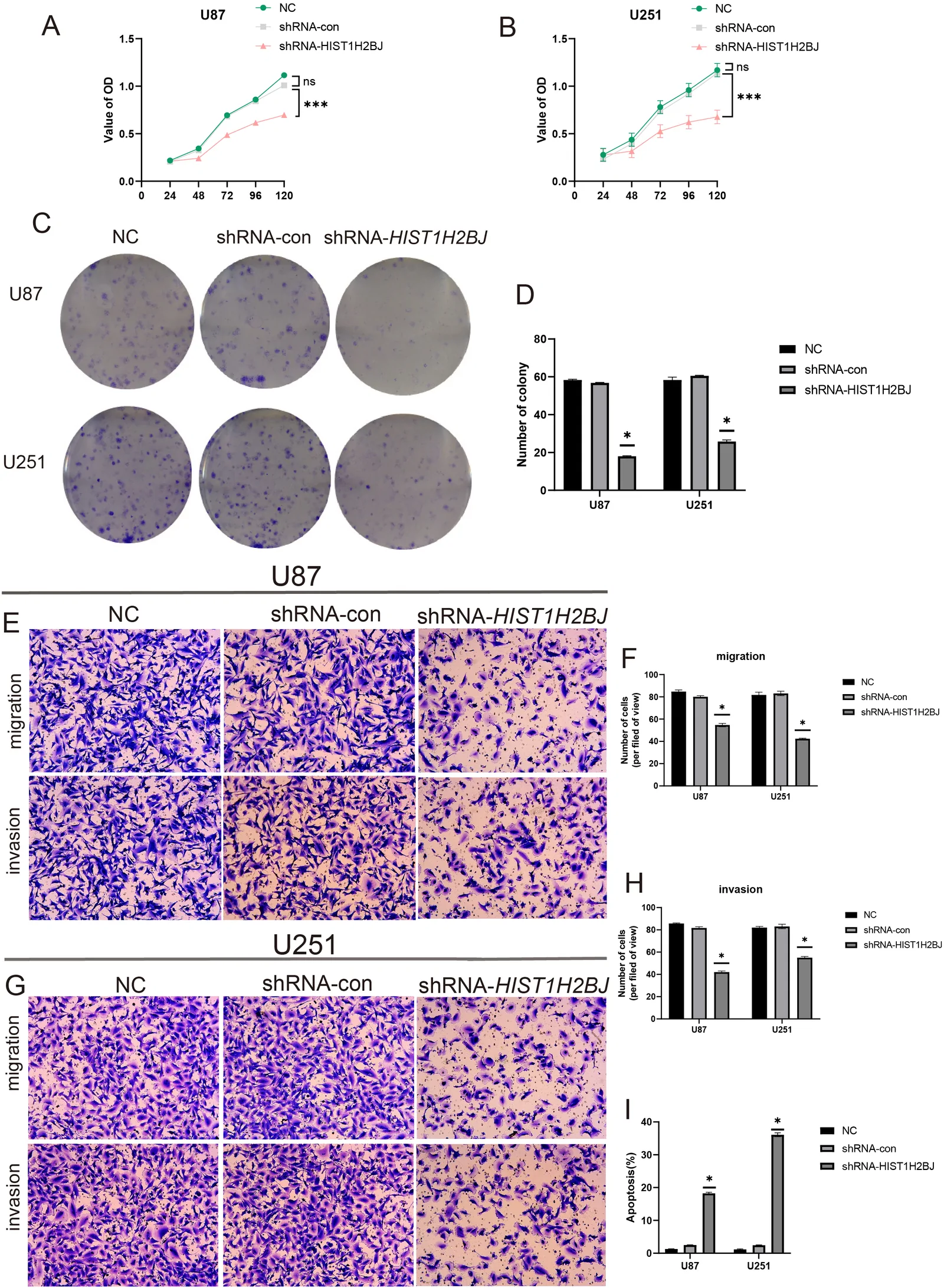
Effects of *HIST1H2BJ* on glioma cell proliferation, migration, invasion, and apoptosis *in vitro*. **(A, B)** The proliferation of U87 and U251 cells with knocked down *HIST1H2BJ* expression was detected by MTT assay. **(C, D)** The proliferation of U87 and U251 cells with knocked down HIST1H2BJ expression was detected by soft agar colony formation assays. **(E–H)** The cell migration and invasion of U87 and U251 cells with *HIST1H2BJ* knocked down by transwell assays. **(I)** The apoptosis of U87 and U251 cells with knocked down *HIST1H2BJ* expression was detected by flow cytometry. NC, normal control; shRNA-con, control scrambled shRNA. Data are presented as mean ± SD from three independent experiments. *p< 0.05, **p< 0.01, ***p< 0.001. ns, not significant (as determined by one-way ANOVA followed by Tukey’s *post-hoc* test).

### 3.4 Analysis of *HIST1H2BJ*-related metabolic characteristics

Metabolic profiling via GSVA of 115 previously reported metabolic-related genes uncovered distinct metabolic dependencies. The *HIST1H2BJ*^high^ exhibited significant enrichment in energy metabolism pathways, including inositol phosphate metabolism, glyoxylate and dicarboxylate metabolism, and pyruvate metabolism. Conversely, the *HIST1H2BJ*^low^ displayed preferential activation of nucleotide precursor pathways, such as amino sugar and nucleotide sugar metabolism, and galactose metabolism (**Figure 4A**). These findings suggest that *HIST1H2BJ* is associated with distinct metabolic reprogramming patterns that may accompany glioma progression.

**Figure 4:**
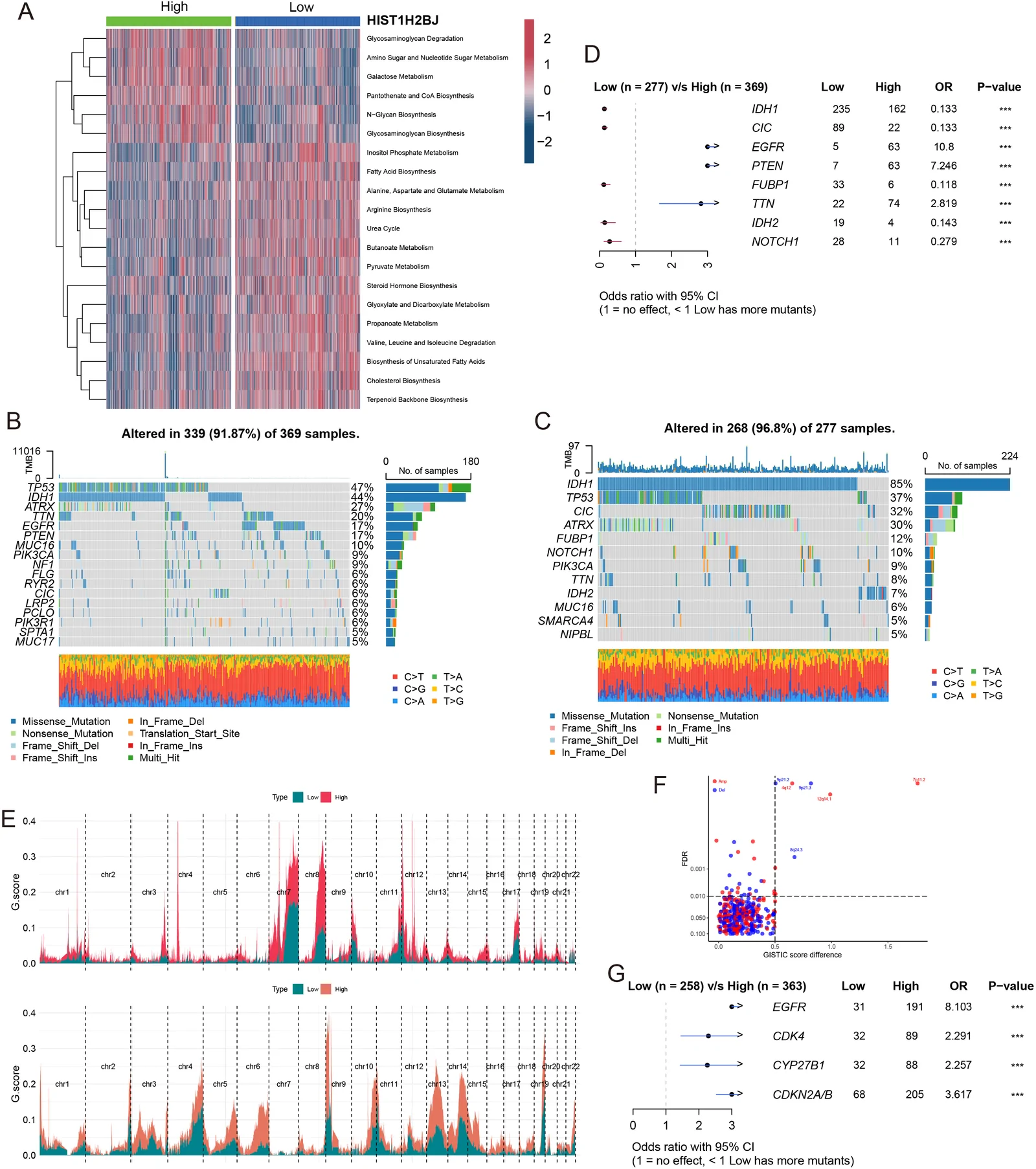
Molecular characteristics of different *HIST1H2BJ* subgroups. **(A)** The heatmaps of the differential enrichments of metabolism-related signatures in the CGGA1 cohorts. **(B, C)** The somatic mutational landscape of the *HIST1H2BJ*^high^ **(B)** and HIST1H2BJlow **(C)** subgroups in the glioma. **(D)** The difference in mutation frequency of high-frequency mutated genes in glioma between the *HIST1H2BJ*^high^ and *HIST1H2BJ*^low^. **(E)** The GISTIC amplification (top) and deletion (bottom) plots of *HIST1H2BJ*^high^ and *HIST1H2BJ*^low^. **(F)** The differentially amplified or deleted regions in *HIST1H2BJ*^low^ compared to *HIST1H2BJ*^high^, with significant regions labeled (FDR< 0.01, and G-score difference > 0.5). **(G)** The difference in the frequency of glioma gene CNV between the *HIST1H2BJ*^high^ and *HIST1H2BJ*^low^. Statistical significance was determined by two-sided Fisher’s exact test; ***p< 0.001.

### 3.5 The association of *HIST1H2BJ* expression with genomic alterations in glioma

To explore the genomic alteration landscape associated with *HIST1H2BJ* expression, we analyzed mutation and CNV data in *HIST1H2BJ*^high^ and *HIST1H2BJ*^low^ gliomas. Mutational profiling detected 17 highly mutated genes in *HIST1H2BJ*^high^ gliomas and 12 in *HIST1H2BJ*^low^ gliomas (mutation frequency ≥5%, **Figures 4B, C**). Comparative analysis revealed differential mutation patterns: *HIST1H2BJ*^high^ gliomas were associated with higher mutation frequencies of *PTEN*, *EGFR*, and *TTN*, whereas *HIST1H2BJ*^low^ gliomas displayed higher mutation frequencies in *IDH* (*IDH1*+*IDH2*), *FUBP1*, *NOTCH1*, and *CIC* (**Figure 4D**).

Somatic copy number alterations (SCNA) analysis revealed differences in 5 genomic regions (9p21.2, 9p21.3, 4q12, 12q14.1, 7p11.2) between *HIST1H2BJ*^high^ and *HIST1H2BJ*^low^ (**Figures 4E, F**). We found that *HIST1H2BJ*^high^ gliomas displayed a higher rate of *EGFR* amplification in the 7p11.2 region, a higher amplification rate of *CYP27B1* and *CDK4* in the 12q14.1 region, and a higher loss rate of *CDKN2A/B* in the 9p21.3 region (**Figure 4G**).

### 3.6 Correlation between *HIST1H2BJ* expression and Immunosuppressive microenvironment in glioma

To understand the role of *HIST1H2BJ* in shaping the glioma TME, we analyzed its correlation with immune score, stromal score, and tumor purity. *HIST1H2BJ* expression showed a prominent negative correlation with tumor purity, while showing strong positive correlations with both stromal score and immune score (**Figure 5A**). These findings suggest that *HIST1H2BJ* overexpression is linked to TME enrichment in stromal and immune components. Further analysis demonstrated significant positive correlations between *HIST1H2BJ* and immune regulatory factors (**Supplementary Figure S7A**). For instance, the expression of Major Histocompatibility Complex (MHC) and chemokines was upregulated in the *HIST1H2BJ*^high^. Collectively, these data suggest that elevated *HIST1H2BJ* is associated with an enhanced antigen processing and presentation machinery in glioma.

**Figure 5:**
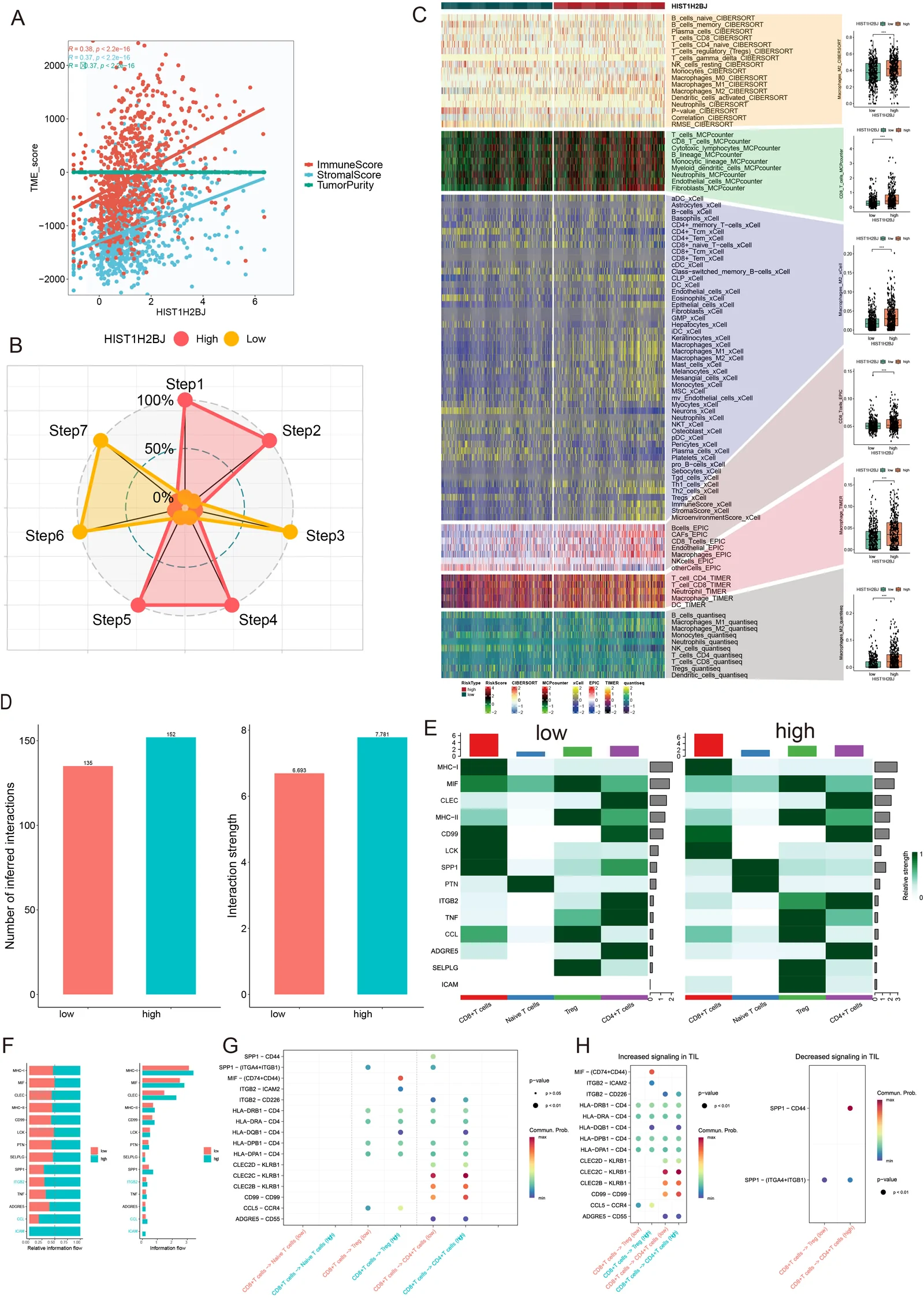
The TME landscapes of glioma patients with different *HIST1H2BJ* subgroups. **(A)** The correlation between *HIST1H2BJ* and immune score, stromal score, and tumor purity. **(B)** The correlation between HIST1H2BJ and anticancer immune activity in the cancer-immune cycle. **(C)** The differences in immune cell infiltration abundance between the *HIST1H2BJ*^high^ and *HIST1H2BJ*^low^. Statistical significance was determined by the Wilcoxon test; ***p< 0.001. **(D)** Bar graphs showing the total number of interactions and interaction strength of cell-to-cell communication network between the *HIST1H2BJ*^high^ and *HIST1H2BJ*^low^. **(E)** Heatmaps showing the overall signaling pathway of each immune cell subpopulation mediated by individual signaling pathway in *HIST1H2BJ*^high^ group (left) and *HIST1H2BJ*^low^ group (right). **(F)** Differences in the overall signaling pathway between the *HIST1H2BJ*^high^ and *HIST1H2BJ*^low^. **(G, H)** Communication probabilities of important ligand-receptor pairs from CD8+T cell to other immune cells in the *HIST1H2BJ*^high^ and *HIST1H2BJ*^low^, with the dot color reflecting the communication probability, blank indicating the communication probability zero, and dot size representing the p value.

To evaluate the functional impact of *HIST1H2BJ* on antitumor immunity, we analyzed the seven-step cancer-immunity cycle using the Tumor Immunophenotype Profiler (TIP). The results revealed higher activity in steps 1 (antigen release), 2 (antigen presentation), 4 (T cells trafficking), and 5 (immune cell infiltration) in the *HIST1H2BJ*^high^, while lower activity in steps 6 (T cell recognition) and 7 (tumor cell apoptosis) (**Figure 5B**). This profile suggests that while *HIST1H2BJ*-high gliomas exhibit active immune recruitment and infiltration, they may develop resistance to terminal effector functions, potentially indicating a state of immune dysfunction where infiltrating T cells fail to effectively recognize and kill tumor cells.

Furthermore, the integration of six immune algorithms revealed distinct immune infiltration patterns associated with *HIST1H2BJ* in glioma patients. The results revealed that *HIST1H2BJ* expression is positively correlated with CD8+ T cells, M2 macrophages, and regulatory T cells (Tregs), while negatively correlating with the infiltration abundance of CD4+ T cells (**Figure 5C**). Notably, *HIST1H2BJ*-high tumors exhibited a significantly positive correlation with immune checkpoint genes such as *PD-1*, *PD-L1*, and *CTLA4* (**Supplementary Figure S7B**).

To explore whether *HIST1H2BJ* expression is associated with altered CD8+ T cell-mediated intercellular communication patterns, we mapped cell-cell communication networks between *HIST1H2BJ*^high^ and *HIST1H2BJ*^low^ gliomas. We found that *HIST1H2BJ*^low^ tumors exhibited reduced network complexity and weaker signaling strength than those in *HIST1H2BJ*^high^ (**Figure 5D**). We also found enhanced CD8+T cell–driver signaling flux, surpassing CD4+T and Treg cells, in both *HIST1H2BJ*^high^ and *HIST1H2BJ*^low^ (**Figure 5E**). In addition, enriched *ICAM*, *CCL*, and *ITGB2* signaling pathways promoted immune cell adhesion and chemotaxis in *HIST1H2BJ*^high^, while enriched MHC-1, MIF, CLEC, and MHC-II signaling pathways indicated altered antigen presentation in *HIST1H2BJ*^low^ (**Figure 5F**).

Because CD8+T cells are key for cell communication in *HIST1H2BJ*^low^ glioma and *HIST1H2BJ*^high^ glioma tissues, and the communication is heterogeneous between tissues, we further compared the probability of ligand-receptor communication between CD8+T and other immune cells in glioma tissues. HLA-DRB1-CD4, HLA-DRA-CD4, HLA-DPB1-CD4, and HLA-DPA1-CD4 are more active in the communication between CD8+ T cells and Treg/CD4+ T cells, which may reflect altered cell-cell information interaction patterns associated with immunotherapy response. The macrophage migration inhibitory factor pathway mediated by mif is more activated in the signal flow from CD8+T cells to Treg cells, and its multi-subunit unit receptor complex CD74-CD44 could coordinate the inflammatory pathway. CLEC2C-KLRB1 signaling pathway was also highly activated in glioma CD8+T cell to CD4+T cell signaling flow (**Figures 5G, H**). CLEC2C-KLRB1 interaction could promote the formation of immunosuppressive microenvironment between memory B cells and KLRB1+PD-1+CD8+ cells. And significantly correlated to the potency of *PD-1* antibody treatment. The above results suggest that CD8+ T cells appear to play a prominent role in intercellular communication networks in gliomas and that communication patterns differ between the two groups.

### 3.7 Correlation of *HIST1H2BJ* expression with immunotherapy response in glioma

We detected a favorable relationship between *HIST1H2BJ* expression and the Tumor Inflammation Signature (TIS) score, T cell co-stimulation level, antigen presentation co-stimulation level, HLA, and other immune markers (**Supplementary Figure S7C**). Additionally, we found that *HIST1H2BJ* is significantly positively correlated with Tumor Mutation Burden (TMB) (**Figure 6A**) and Tumor Neoantigen Burden (TNB) (**Figure 6B**), while negatively correlated with Intra-Tumoral Heterogeneity (ITH) (**Figure 6C**). Given that lower ITH is often associated with improved immunotherapy outcomes, this inverse correlation may further contribute to therapeutic efficacy.

**Figure 6:**
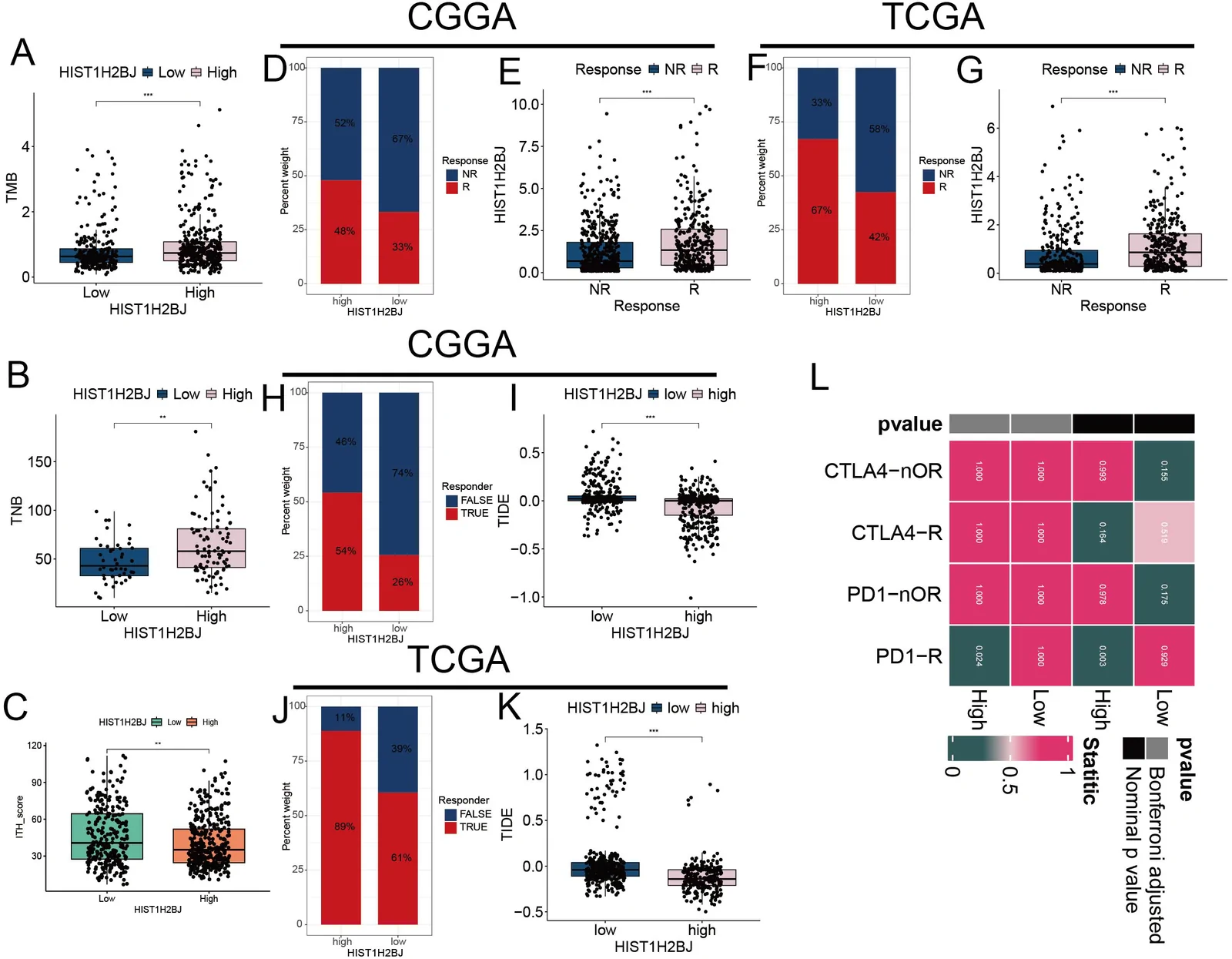
The immunotherapy response landscapes of glioma patients with different *HIST1H2BJ* subgroups. **(A-C)** The differences in **(A)**TMB, **(B)**TNB, and **(C)** ITH_score between the *HIST1H2BJ*^high^ and *HIST1H2BJ*^low^. Statistical significance was determined by the Wilcoxon test; **p< 0.01, ***p< 0.001. **(D–G)** The association between *HIST1H2BJ* and the computationally predicted immunotherapy response of glioma patients in **(D, E)** CGGA and **(F, G)** TCGA datasets by ImmuCellAI. Statistical significance was determined by the Wilcoxon test; ***p< 0.001. **(H–K)** The correlation between TIDE score of glioma patients and *HIST1H2BJ* expression in CGGA and TCGA datasets. **(L)** The submap analysis of *HIST1H2BJ* expression and computationally predicted immunotherapy response in CGGA. P-values were obtained after being adjusted.

To explore the potential association between *HIST1H2BJ* expression and immunotherapy response, we used the ImmuCellAI and TIDE algorithms to computationally assess whether *HIST1H2BJ*^high^ and *HIST1H2BJ*^low^ differed in their predicted response to immune checkpoint blockade. Both algorithms suggested that *HIST1H2BJ*^high^ gliomas had a higher computationally predicted likelihood of response to immune checkpoint inhibitors than *HIST1H2BJ*^low^ gliomas (**Figures 6D–K**). SubMap analysis further predicted the therapeutic response of glioma patients to anti-CTLA4 and anti-PD-1 immunotherapy (**Figure 6L**). Collectively, these results suggest that *HIST1H2BJ* may be associated with computationally predicted sensitivity to anti-PD-1 immunotherapy in glioma; this association is hypothesis-generating and requires validation in glioma patients treated with immune checkpoint inhibitors.

### 3.8 Prognostic predictive performance of IMPMG in glioma

We constructed the Immune-Metabolic Prognostic Model of Glioma (IMPMG) by integrating *HIST1H2BJ* expression, immune-metabolic genes score (IMGscore), and clinical variables (age, tumor grade, IDH status) through stepwise multivariate Cox regression (**Supplementary Figure S8**). Patients were stratified into high- and low-IMPMG group based on median risk scores. KM survival curves across CGGA1, CGGA2, and TCGA datasets consistently demonstrated significantly shorter OS in high-IMPMG patients versus low-IMPMG counterparts (**Figures 7A–C**). The IMPMG model exhibited robust accuracy (all AUC > 0.86, **Figures 7D–F**). Calibration curve analysis confirmed strong agreement between predicted and observed survival probabilities (**Supplementary Figures S9A–C**). Compared to *HIST1H2BJ*, IMPMG model improved predictive performance by 13% in 2-year AUC, 14% in 3-year AUC, and 13% in 5-year AUC. Similarly, IMPMG outperformed IMGscore with relative AUC increases of 4%, 5%, and 2% respectively (**Supplementary Figures S9D–F**). Moreover, C-index values further validated IMPMG’s superiority, achieving values significantly higher than *HIST1H2BJ* and IMGscore (**Supplementary Figures S9G–I**).

**Figure 7:**
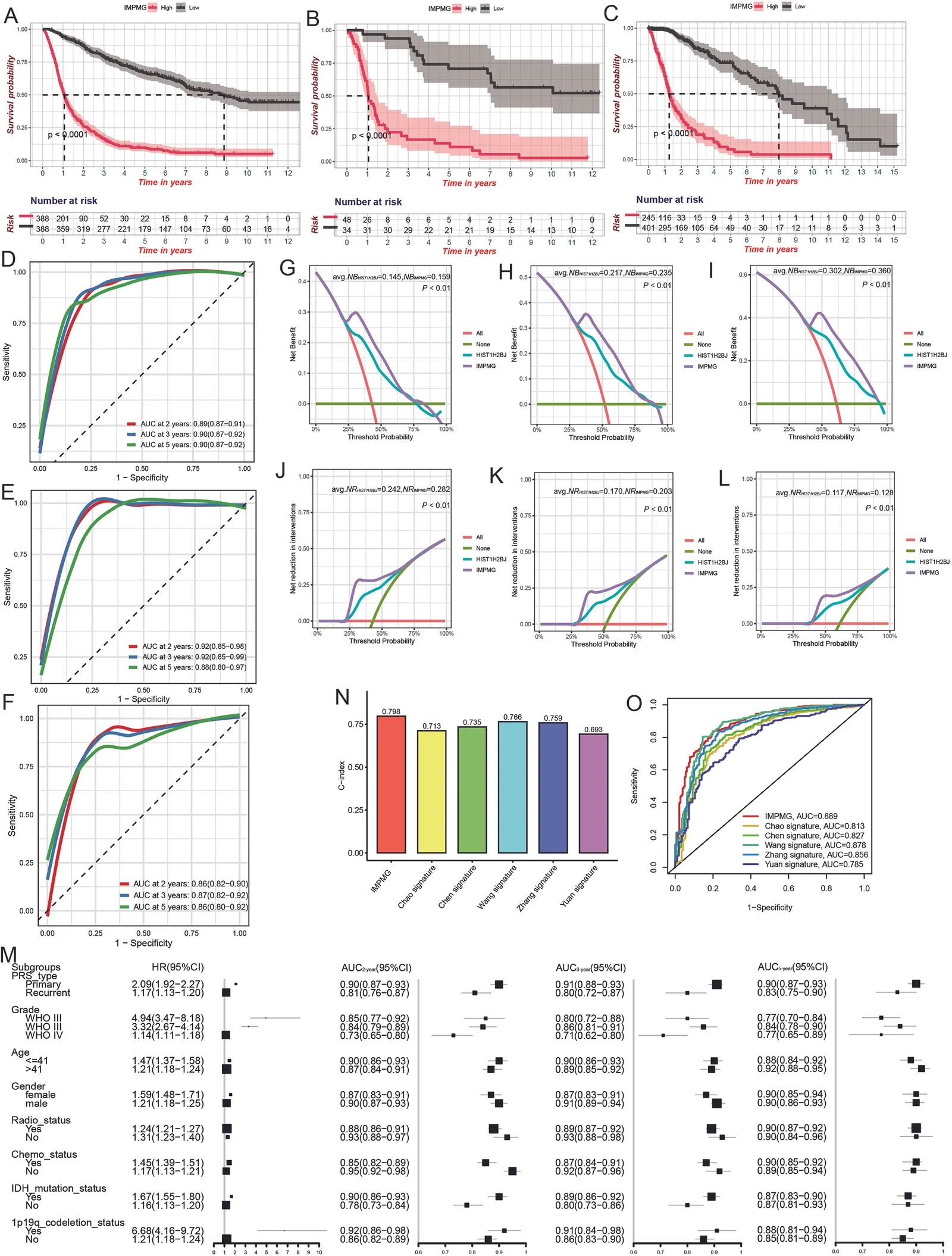
Assessment of predictive performance of IMPMG on glioma patient prognosis. **(A-C)** The survival differences between high- and low-IMPMG patients in **(A)** CGGA1, **(B)** CGGA2 and **(C)** TCGA cohorts. Patients in all 3 cohorts were categorized into two groups based on the same cutoff point: the median of IMPMG score defined in CGGA1 training set. The log-rank test was used to calculate the p-value. **(D–F)** The time-dependent ROC and AUC of IMPMG in **(D)** CGGA1, **(E)** CGGA2 and **(F)** TCGA cohorts, respectively. **(G-L)** The decision Curve Analysis (DCA) evaluating the net benefit (NB) and net reduction (NR) for patients at 2-year **(G, J)**, 3-year **(H, K)**, and 5-year **(I, L)** survival intervals. **(M)** The survival analysis of 2-year AUC, 3-year AUC, and 5-year AUC in different clinical subgroups of glioma patients. **(N, O)** The comparison of C-index **(N)** and AUC **(O)** in predicting prognosis between IMPMG and other models for glioma.

To further quantify the incremental predictive value of IMPMG beyond established clinical variables, we compared IMPMG with a clinical-variables-only baseline model using the integrated discrimination improvement (IDI) and net reclassification improvement (NRI). In the CGGA1 training cohort, IMPMG improved risk discrimination at the 2-year survival endpoint, with an IDI of 0.029 (95% CI: 0.007–0.052, p = 0.008) and an NRI of 0.233 (95% CI: 0.000–0.337, p = 0.050), the latter reaching borderline significance. This incremental value persisted at 3-year survival (IDI = 0.019, 95% CI: 0.001–0.040, p = 0.038; NRI = 0.213, 95% CI: 0.030–0.306, p = 0.018) and 5-year survival (IDI = 0.034, 95% CI: 0.011–0.058, p = 0.004; NRI = 0.273, 95% CI: 0.014–0.365, p = 0.038), confirming that IMPMG provides prognostic information beyond classical clinical predictors (**Supplementary Table S7**).

To evaluate the clinical utility of the IMPMG prognostic model, we performed decision curve analysis (DCA) across 2-year, 3-year, and 5-year survival endpoints. Specifically, at the 2-year survival endpoint, IMPMG achieved a higher net benefit (NB = 0.161) than *HIST1H2BJ* (NB = 0.10). Because net benefit is a threshold-weighted quantity rather than a direct count of patients, this difference should be interpreted as a higher standardized net benefit at the corresponding threshold probability, not as a literal number of additional patients who benefit. This advantage persisted at the 3-year and 5-year survival endpoints, with IMPMG consistently maintaining higher net benefits (**Figures 7G–I**). Furthermore, IMPMG exhibited higher net reduction (NR) in false-positive interventions across all timepoints, underscoring its precision in risk stratification (**Figures 7J–L**). Sensitivity analysis confirmed IMPMG’s consistent superiority over *HIST1H2BJ* and baseline strategies across a wide range of clinical decision thresholds.

To assess the reliability of IMPMG in predicting the prognosis of glioma patients, we conducted a subgroup analysis across various clinical characteristics, including PRS_type, age, Grade, Gender, Radio_status, Chemo_status, IDH_mutation_status, and 1p19q_codeletion_status. In all analyzed subgroups, IMPMG maintained robust performance across all cohorts, demonstrating consistent performance across the analyzed clinical subgroups (**Figure 7M**). In head-to-head comparisons with five published prognostic models (29–33), IMPMG achieved superior discriminative ability in the CGGA1 dataset, with significant gains in both C-index and AUC (**Figures 7N, O**).

### 3.9 Drug sensitivity analysis and molecular docking

Current chemotherapy regimens for glioma remain suboptimal, underscoring the urgent need for novel therapeutic strategies. Our findings indicate that *HIST1H2BJ* is associated with glioma malignancy and poor patient prognosis. To explore potential therapeutic relevance, we employed the pRRophetic algorithm to predict drug sensitivity based on *HIST1H2BJ* expression profiles. This analysis identified two compounds, VX_680 and A.770041, which exhibited significantly lower predicted IC50 values in the *HIST1H2BJ*^high^ (**Figure 8A**). Furthermore, molecular docking suggested potential binding interactions between *HIST1H2BJ* and both compounds, with binding energies of -7.6 kcal/mol and -7.3 kcal/mol for VX_680 and A.770041, respectively. The primary interactions involved Pi-alkyl interactions, hydrogen bonds, and Pi-Sigma interactions between *HIST1H2BJ* residues and the ligands (**Figures 8B, C**).

**Figure 8:**
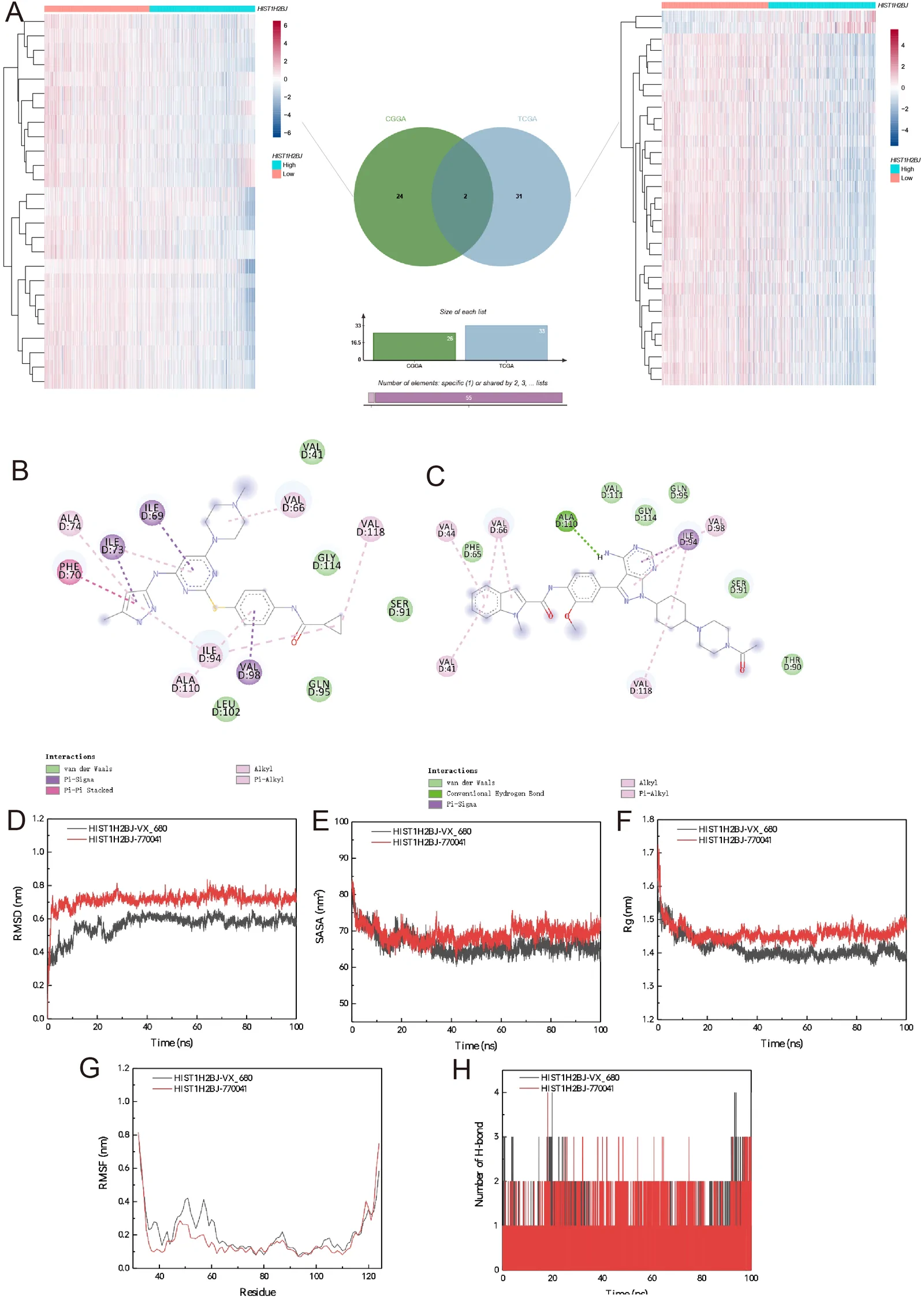
Molecular docking and molecular dynamics simulation of two small-molecule compounds. **(A)** The difference in IC50 of small-molecule compounds between the *HIST1H2BJ*^high^ and the *HIST1H2BJ*^low^. **(B, C)** The docking results of HIST1H2BJ-VX_680 **(B)** and the docking results of HIST1H2BJ-A.770041 **(C)**. **(D)** RMSD of two systems. **(E)** SASA of two systems. **(F)** Rg of two systems. **(G)** RMSF of two systems. **(H)** Number of hydrogen bonds in two systems.

Molecular dynamics simulations further suggested the stability of these complexes. Root mean square deviation (RMSD) analysis indicated that the HIST1H2BJ-VX_680 complex stabilized at 0.56 ± 0.07 nm after 10 ns, while the HIST1H2BJ-A.770041 complex reached stability at 0.71 ± 0.05 nm by 30 ns (**Figure 8D**). Root mean square fluctuation (RMSF) analysis showed backbone fluctuations of ≤ 0.21 nm and 0.18 nm, respectively, suggesting minimal conformational disruption upon ligand binding (**Figure 8G**). Although the average number of hydrogen bonds was relatively low (0.71 for VX_680 and 0.68 for A.770041), intermittent hydrogen bonding was observed throughout the simulation. Furthermore, both solvent-accessible surface area (SASA) and radius of gyration (Rg) values remained stable at relatively low levels, indicating that the protein maintains a compact structure when bound to the ligands (**Figures 8D–H**). Consistent with the docking results, MM-PBSA calculations yielded total binding free energies of -439.54 kJ/mol for VX_680 and -300.95 kJ/mol for A.770041 (**Supplementary Table S8**), indicating strong binding affinity driven largely by van der Waals forces.

## 4 Discussion

Glioma’s pronounced heterogeneity and therapy resistance have rendered conventional treatments largely ineffective, with minimal improvement in patient outcomes over decades (34–37). While existing biomarkers, such as *IDH* mutations, provide limited prognostic stratification, the absence of clinically actionable biomarkers hinders personalized treatment strategies. Through integrative single–cell analysis and bulk transcriptomic profiling, we observed that elevated expression of the histone-coding gene *HIST1H2BJ* correlates with reduced overall survival (OS) in glioma patients. Elevated *HIST1H2BJ* expression is also associated with an immunosuppressive tumor microenvironment marked by M2 macrophage infiltration, Treg expansion, and CD8+T cell dysfunction. Subsequently, we identified immune- and metabolic-related genes correlated with *HIST1H2BJ* and developed the IMPMG prognostic model, which demonstrated strong predictive accuracy in both the CGGA and TCGA datasets. Furthermore, decision curve analysis indicated that IMPMG provides favorable clinical net benefits for identifying high-risk glioma patients.

As *HIST1H2BJ* is a core histone-coding gene, it is intrinsically involved in chromatin remodeling and transcriptional regulation. Chromatin remodeling—a key upstream regulatory mechanism governing histone modification—is intrinsically linked to the dysregulation of gene expression in cancer. Dysfunctional chromatin remodelers (CRs) disrupt nucleosome-DNA interactions, driving aberrant epigenetic modifications (38–42). Furthermore, genetic alteration analysis revealed differential somatic mutation and CNV profiles between *HIST1H2BJ*^high^ and *HIST1H2BJ*^low^ gliomas. Specifically, *HIST1H2BJ*^high^ tumors were characterized by a higher frequency of *EGFR* mutations, which are known to be associated with constitutive *EGFR* activation and uncontrolled proliferation (43, 44), while *HIST1H2BJ*^low^ gliomas showed enrichment of prognostically favorable *IDH* mutations (6). These patterns are consistent with the established genomic distinction between IDH-wildtype and IDH-mutant gliomas, and may therefore be influenced by underlying molecular subtype. Functional validation confirmed that, *in vitro*, *HIST1H2BJ* knockdown significantly suppressed glioma malignancy by inhibiting cell proliferation and migration, as well as reducing invasion capacity. Collectively, these findings suggest that *HIST1H2BJ* is associated with genomic features commonly observed in aggressive gliomas, possibly reflecting its participation in chromatin dysregulation.

Gliomas are widely recognized as immunologically “cold” tumors due to their limited immune cell infiltration and poor response to immune checkpoint inhibitors (ICIs), such as *CTLA-4* and *PD-1*, which have demonstrated minimal efficacy in clinical trials (45–47). Consequently, targeting these immune checkpoints has failed to yield significant clinical benefits in glioma patients (48). In our study, we discovered that elevated *HIST1H2BJ* expression correlates significantly with stromal and immune scores, while inversely associating with tumor purity. Additionally, *HIST1H2BJ*-high gliomas exhibit reduced infiltration of naive CD4+ T cells and monocytes, but an increased infiltration of immunosuppressive M2 macrophages, Tregs, and CD8+ T cells. Within the glioma microenvironment, tumor-associated macrophages (TAMs) and myeloid-derived suppressor cells (MDSCs) adopt immunosuppressive programs, secreting pro-tumorigenic cytokines that foster angiogenesis and immune evasion (49, 50). Integrative single-cell and spatial analyses have further resolved TAMs into distinct subpopulations—including hypoxic macrophages with vasculature-normalizing potential and antigen-presenting subsets that drive T-cell exhaustion—highlighting the spatial and functional heterogeneity of myeloid compartments in glioma (51). Despite the increased infiltration of CD8+ T cells, this immunosuppressive milieu likely drives them into a dysfunctional or exhausted state. This paradox is further corroborated by our cancer-immunity cycle analysis, which revealed impaired T cell activity in *HIST1H2BJ*-high gliomas, particularly in tumor antigen recognition (step 6) and tumor cell apoptosis (step 7). Importantly, these observations are best interpreted as an “inflamed but exhausted,” checkpoint-rich phenotype rather than a simple immune-excluded state. Although this immunosuppressed, checkpoint-high microenvironment underlies the poor prognosis of *HIST1H2BJ*-high gliomas, several of the same features—elevated immune-checkpoint expression, abundant CD8+ T-cell infiltration, high tumor mutational burden, and low intratumoral heterogeneity—are also recognized predictors of responsiveness to immune checkpoint blockade. This duality may reconcile the apparent contradiction between the immunosuppressive microenvironment and the favorable immunotherapy response that is computationally predicted for *HIST1H2BJ*-high gliomas, a prediction that nonetheless requires validation in immunotherapy-treated cohorts. Mechanistically, the concurrent expansion of M2 macrophages and regulatory T cells and the up-regulation of *PD-1*, *PD-L1*, and *CTLA-4* are likely to promote immune evasion despite abundant T-cell infiltration, so the TIDE and ImmuCellAI predictions should be regarded only as hypotheses.

Intercellular communication among tumor microenvironment components is a critical driver of tumor progression and strongly dictates the efficacy of therapeutic interventions. To elucidate the underlying mechanisms, we performed cell-cell communication analysis to identify the most interactive cell subpopulations. This analysis highlighted CD8+ T cells as central interactors; however, these interactions were highly heterogeneous, with *HIST1H2BJ*^low^ gliomas displaying significantly attenuated communication networks compared to *HIST1H2BJ*^high^ gliomas. Collectively, these findings underscore that elevated *HIST1H2BJ* expression in gliomas is associated with enhanced intercellular communication and an immunosuppressive phenotype, characterized by the upregulation of immune checkpoints, cytokine dysregulation, and the infiltration of immunosuppressive cells. This microenvironment may be associated with poor patient prognosis and resistance to immunotherapies.

Our research revealed a marked positive correlation between *HIST1H2BJ* expression and key immunotherapy-related biomarkers, including HLA expression, TIS scores, and TMB. These findings align with prior research emphasizing the critical role of T cell co-stimulatory signaling pathways and antigen presentation in driving effective anti-tumor immune responses (52). Specifically, elevated HLA and TIS scores have been associated with heightened sensitivity to immune checkpoint inhibitors, particularly anti-PD-L1 treatments (25, 53). Moreover, high TMB has been linked to increased neoantigen production, which enhances T-cell recognition and improves outcomes with immune checkpoint blockade (54). Consistently, both ImmuCellAI and TIDE algorithms predicted that *HIST1H2BJ*^high^ gliomas are more likely to respond to immunotherapy. This prediction was further supported by Submap analysis, which revealed a significant association between high *HIST1H2BJ* expression and favorable immunotherapy responses. Collectively, these findings suggest that HIST1H2BJ may serve as a potential immune-response-associated signature related to immunotherapy efficacy in glioma patients, although validation in immunotherapy-treated cohorts is warranted.

To translate these findings into clinical utility, we moved beyond mere prognostication to explore actionable therapeutic strategies. First, we developed the IMPMG model to refine clinical decision-making. By synergizing *HIST1H2BJ* expression with immune-metabolic genes and key clinical variables (age, tumor grade, IDH status), this model offers a robust tool for precision prognostication and personalized management. However, accurate stratification must be paired with effective intervention. Therefore, to identify candidate compounds whose predicted drug sensitivity differed by *HIST1H2BJ* expression, we combined drug-sensitivity prediction with exploratory molecular docking, which nominated two compounds, VX-680 and A.770041. It should be emphasized, however, that these findings are hypothesis-generating. Core histone proteins are structural components of the nucleosome that are tightly wrapped by DNA, and they therefore lack the conventional, druggable binding pockets typically engaged by small molecules. Moreover, VX-680 is an established Aurora kinase inhibitor and A.770041 is a selective Lck inhibitor, and the docking was performed against an AlphaFold-predicted monomeric structure. The in silico binding energies therefore do not imply functional inhibition of *HIST1H2BJ* itself and may instead reflect secondary binding sites or overlapping structural motifs; experimental studies are required to determine whether these compounds modulate *HIST1H2BJ* function or expression in glioma cells.

This study has several limitations. First, our conclusions are based largely on retrospective public cohorts (CGGA, TCGA, and GEO), and most immune and metabolic findings derive from bulk deconvolution, ssGSEA, and cell-cell communication inference, which demonstrate association rather than causation. Because *HIST1H2BJ* expression is strongly correlated with tumor grade, IDH status, and 1p/19q codeletion, the observed immune and prognostic associations may be partly confounded by glioma subtype and malignant progression; *HIST1H2BJ* may therefore act, at least in part, as a surrogate marker of aggressive biology rather than an independent driver, and its incremental prognostic value beyond established markers warrants more rigorous evaluation. Second, the IMPMG model incorporates strong clinical and molecular predictors (age, grade, IDH and 1p/19q status), so its high accuracy may be driven substantially by these variables; comparison against a clinical-only baseline model, internal cross-validation or bootstrap correction, and validation in truly independent cohorts are needed to establish its added value. Third, the machine-learning feature-selection pipeline used survival information and may be susceptible to selection bias and overfitting; the detailed procedures are provided in the supplementary materials and should be interpreted with this caveat. Fourth, the *in vitro* experiments support a tumor-promoting role for *HIST1H2BJ* but do not define its mechanism, as rescue (for example, re-expression of an shRNA-resistant construct), overexpression, pathway-level, and *in vivo* (orthotopic) experiments were not performed; in addition, because *HIST1H2BJ* is a core histone, its knockdown may exert broad transcriptional effects that the present data cannot fully exclude, and the key cell line U87 has recognized limitations in representing primary glioblastoma biology, so validation in patient-derived or glioma stem-like cultures is warranted. Fifth, promoter hypomethylation was associated with *HIST1H2BJ* overexpression but was not validated experimentally (for example, by bisulfite sequencing, methylation-specific PCR, or treatment with demethylating agents). Sixth, the predicted responses to immune checkpoint blockade rely on computational tools (ImmuCellAI, TIDE, and submap analysis) that were largely developed in other tumor types and may not transfer to the distinctive glioma immune context; these predictions require validation in glioma patients treated with immune checkpoint inhibitors. Seventh, the candidate compounds VX_680 and A.770041 were nominated by drug-sensitivity prediction and docking to an AlphaFold-predicted structure; because *HIST1H2BJ* is a histone rather than a conventional drug target and these compounds are known kinase inhibitors, this analysis should be regarded as preliminary hypothesis generation pending experimental validation. Finally, the clinical specimen cohort was small (n = 27 gliomas and n = 5 non-tumor tissues), so the histological findings are preliminary and should be confirmed in larger cohorts with paired molecular and survival data.

## 5 Conclusion

Our study identifies *HIST1H2BJ* as a biomarker associated with poor prognosis and an immune-altered microenvironment in glioma. By integrating *HIST1H2BJ* expression with immune-metabolic signatures and established clinical variables, the IMPMG model may aid risk stratification, although its incremental value over established molecular and clinical predictors requires further evaluation. The associations with computationally predicted immunotherapy response and the candidate small-molecule compounds identified here are based on computational analyses and should be regarded as hypothesis-generating, requiring experimental and clinical validation before any therapeutic targeting of HIST1H2BJ could be considered.

## Data Availability

Publicly available datasets were analyzed in this study. This data can be found here: The RNA-seq data of CGGA and TCGA were separately from http://www.cgga.org.cn/ and https://portal.gdc.cancer.gov/.
