# Supplementary material for: HIST1H2BJ is associated with poor prognosis and an immune-altered tumor microenvironment in glioma
Source: Front Immunol. 2026 Sep 16;17:1929885. doi: 10.3389/fimmu.2026.1929885 (PMC13624164; doi:10.3389/fimmu.2026.1929885)
Supplement: Supplementary file 1 [file DataSheet1.pdf]

附件 5

潍坊市中医院医学科研伦理审查批件

伦理批号：2024YX138

<table>
<tr><td>申请原因</td><td colspan="5">☑ 项目申报 ☐论文发表 ☐成果报奖 ☐其他（请注明）______</td></tr>
<tr><td colspan="6">项目基本信息</td></tr>
<tr><td>项目名称</td><td colspan="5">免疫治疗相关心脏损伤的全程化中医药优势方案研究-课题 2：免疫治疗相关心脏损伤预测模型及分级诊断标准建立</td></tr>
<tr><td>项目状态</td><td colspan="5">☐申请中 ☑实施中 ☐已完成</td></tr>
<tr><td>研究进展</td><td colspan="5">☑ 尚未开始 ☐正在进行 ☐已经完成</td></tr>
<tr><td>起止时间</td><td colspan="5">2024 年 08 月——2028 年 07 月</td></tr>
<tr><td>项目类别</td><td colspan="5">☐新药物临床试验 ☐医疗器械临床试验 ☐新技术应用 ☐人体标本收集
☑ 其他（请注明）： 多中心观察性研究</td></tr>
<tr><td>经费来源</td><td colspan="5">☑ 纵向（中央/地方财政资金资助）科技创新 2030—“癌症、心脑血管、呼吸和代谢性疾病防治研究”重大项目（2023ZD0502802）
☐横向（社会资金资助）______</td></tr>
<tr><td>样本类型</td><td colspan="5">☐健康者 ☑ 病人 ☐其他______
☑ 组织样本 ☑ 血、尿样本 ☐其他 ______</td></tr>
<tr><td colspan="6">申请人（项目负责人）基本信息</td></tr>
<tr><td>姓 名</td><td>刘瑞娟</td><td>性 别</td><td>女</td><td>学 历</td><td>硕士研究生</td></tr>
<tr><td>职 称</td><td>副主任医师</td><td>职 务</td><td>科副主任</td><td>科 室</td><td>肿瘤科</td></tr>
<tr><td>办公电话</td><td>0536-8590803</td><td>手 机</td><td>15964583192</td><td>电子邮箱</td><td>zhongliuyike@163.com</td></tr>
<tr><td colspan="6">申请人承诺：
以上所填内容均属实，如获批准，我将严格按照提供的方案进行研究，并遵守潍坊市中医院医学科研伦理委员会的相关规定。
申请人签字：刘瑞娟
年 月 日</td></tr>
<tr><td colspan="6">申报人所在科室意见（必须填写）：
科主任签字：庞静
年 月 日</td></tr>
<tr><td colspan="6">医学科研伦理委员会审批意见：
该研究中受试者权力和利益得到充分保护，对受试者不存在潜在风险，同意该项研究的现场工作按计划进行。
医学科研伦理委员会（盖章）
年 月 日</td></tr>
</table>

注：本表 A4 纸打印，一式两份，不可附页。

潍坊市中医院 医学科研伦理委员会

扫描全能王
3亿人都在用的扫描App
