# Supplementary material for: HIST1H2BJ is associated with poor prognosis and an immune-altered tumor microenvironment in glioma
Source: Front Immunol. 2026 Sep 16;17:1929885. doi: 10.3389/fimmu.2026.1929885 (PMC13624164; doi:10.3389/fimmu.2026.1929885)
Supplement: Supplementary file 2 [file DataSheet2.pdf]

Appendix 5

WEIFANG HOSPITAL OF TRADITIONAL CHINESE MEDICINE
MEDICAL RESEARCH ETHICS REVIEW APPROVAL

Ethics Approval No.: 2024YX138

**Purpose of Application**	☑ Project Application □ Manuscript Publication □ Research Achievement Award □ Other (please specify)
**PROJECT INFORMATION**	
**Project Title**	Research on an Optimized Full-Course Traditional Chinese Medicine Regimen for Immunotherapy-Related Cardiac Injury — Subproject 2: Development of a Prediction Model and Graded Diagnostic Criteria for Immunotherapy-Related Cardiac Injury
**Project Status**	□ Application Pending ☑ In Progress □ Completed
**Study Progress**	☑ Not Yet Started □ Ongoing □ Completed
**Study Period**	August 2024 – July 2028
**Project Type**	□ New Drug Clinical Trial □ Medical Device Clinical Trial □ New Technology Application □ Human Specimen Collection<br>☑ Other (please specify): Multicenter Observational Study
**Funding Source**	☑ Government-Funded Project (central/local fiscal support): Science and Technology Innovation 2030 — Major Project on the Prevention and Control of Cancer, Cardiovascular and Cerebrovascular Diseases, Respiratory Diseases, and Metabolic Diseases (2023ZD0502802)<br>□ Contract-Funded Project (social funding)
**Participant / Specimen Type**	□ Healthy Volunteers ☑ Patients □ Other<br>☑ Tissue Specimens ☑ Blood and Urine Specimens □ Other

APPLICANT (PRINCIPAL INVESTIGATOR) INFORMATION

**Name**	Liu Ruijuan	**Sex**	Female	**Educational Background**	Master's Degree
**Professional Title**	Associate Chief Physician	**Position**	Deputy Department Director	**Department**	Oncology
**Office Telephone**	0536-8590803	**Mobile Phone**	15964583192	**Email**	zhongliuyike@163.com

Applicant's Declaration:

I hereby declare that all information provided above is true and accurate. If approved, I will conduct the research strictly in accordance with the submitted protocol and comply with the relevant regulations of the Medical Research Ethics Committee of Weifang Hospital of Traditional Chinese Medicine.

Applicant's Signature:
Date: ______ / ______ / ______

Comments of the Applicant's Department (Required):

Department Director's Signature:
Date: ______ / ______ / ______

Medical Research Ethics Committee Decision:

The rights and interests of the participants in this study are adequately protected, and no foreseeable potential risks have been identified. The on-site activities of this study are approved to proceed as planned.

Medical Research Ethics Committee (Official Seal)
Date: ______ / ______ / ______

Note: This form shall be printed on A4 paper in duplicate. No additional pages may be attached.
