# Supplementary material for: HIST1H2BJ is associated with poor prognosis and an immune-altered tumor microenvironment in glioma
Source: Front Immunol. 2026 Sep 16;17:1929885. doi: 10.3389/fimmu.2026.1929885 (PMC13624164; doi:10.3389/fimmu.2026.1929885)
Supplement: Supplementary file 3 [file Image3.pdf]

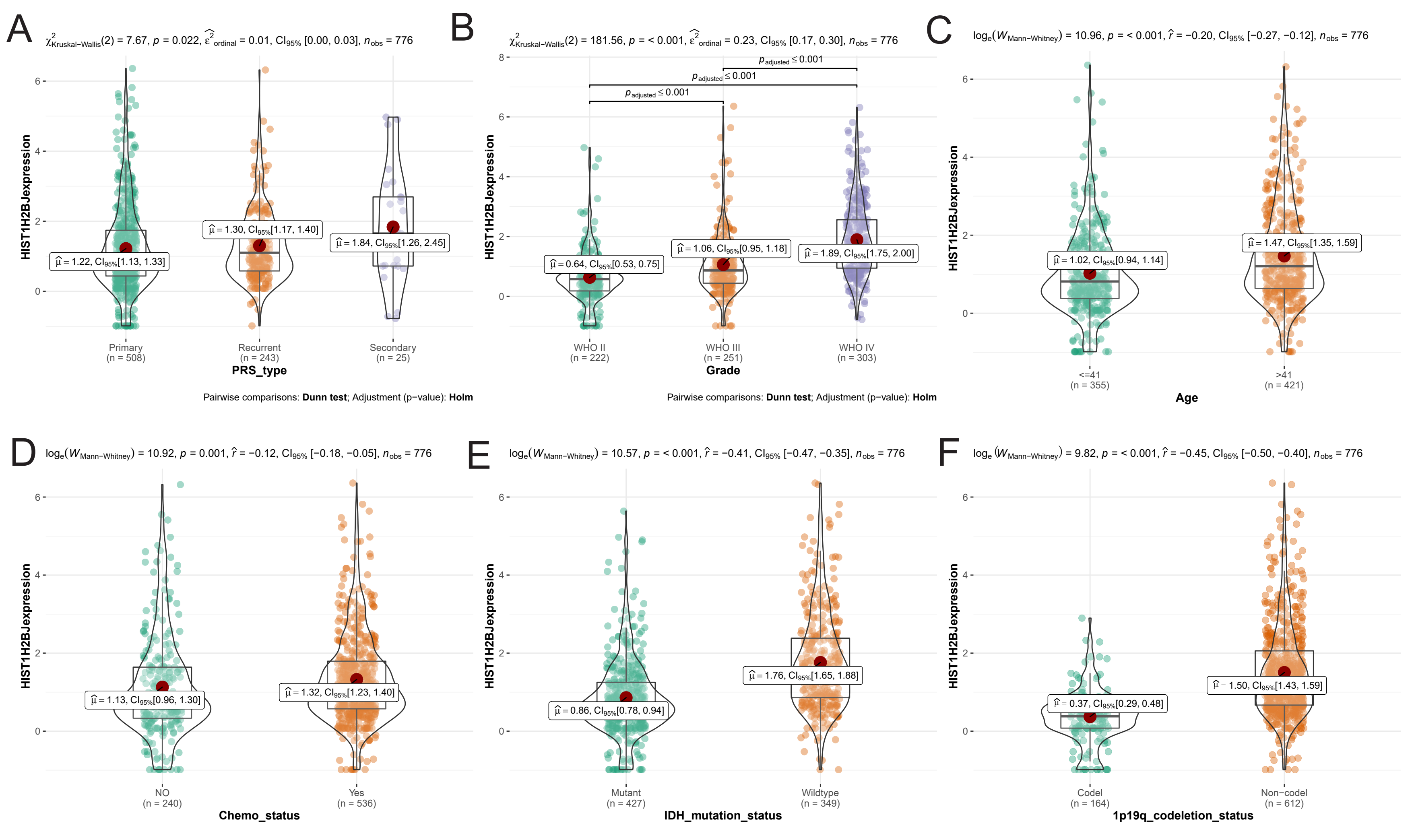
A
χ²Kruskal–Wallis(2) = 7.67, p = 0.022, ε²ordinal = 0.01, CI95% [0.00, 0.03], nobs = 776
HIST1H2BJexpression
μ̂ = 1.22, CI95%[1.13, 1.33]
μ̂ = 1.30, CI95%[1.17, 1.40]
μ̂ = 1.84, CI95%[1.26, 2.45]
Primary (n = 508)
Recurrent (n = 243)
Secondary (n = 25)
PRS_type
Pairwise comparisons: Dunn test; Adjustment (p-value): Holm
B
χ²Kruskal–Wallis(2) = 181.56, p = < 0.001, ε²ordinal = 0.23, CI95% [0.17, 0.30], nobs = 776
padjusted ≤ 0.001
padjusted ≤ 0.001
padjusted ≤ 0.001
HIST1H2BJexpression
μ̂ = 0.64, CI95%[0.53, 0.75]
μ̂ = 1.06, CI95%[0.95, 1.18]
μ̂ = 1.89, CI95%[1.75, 2.00]
WHO II (n = 222)
WHO III (n = 251)
WHO IV (n = 303)
Grade
Pairwise comparisons: Dunn test; Adjustment (p-value): Holm
C
loge(WMann–Whitney) = 10.96, p = < 0.001, r̂ = −0.20, CI95% [−0.27, −0.12], nobs = 776
HIST1H2BJexpression
μ̂ = 1.02, CI95%[0.94, 1.14]
μ̂ = 1.47, CI95%[1.35, 1.59]
<=41 (n = 355)
>41 (n = 421)
Age
D
loge(WMann–Whitney) = 10.92, p = 0.001, r̂ = −0.12, CI95% [−0.18, −0.05], nobs = 776
HIST1H2BJexpression
μ̂ = 1.13, CI95%[0.96, 1.30]
μ̂ = 1.32, CI95%[1.23, 1.40]
NO (n = 240)
Yes (n = 536)
Chemo_status
E
loge(WMann–Whitney) = 10.57, p = < 0.001, r̂ = −0.41, CI95% [−0.47, −0.35], nobs = 776
HIST1H2BJexpression
μ̂ = 0.86, CI95%[0.78, 0.94]
μ̂ = 1.76, CI95%[1.65, 1.88]
Mutant (n = 427)
Wildtype (n = 349)
IDH_mutation_status
F
loge(WMann–Whitney) = 9.82, p = < 0.001, r̂ = −0.45, CI95% [−0.50, −0.40], nobs = 776
HIST1H2BJexpression
μ̂ = 0.37, CI95%[0.29, 0.48]
μ̂ = 1.50, CI95%[1.43, 1.59]
Codel (n = 164)
Non−codel (n = 612)
1p19q_codeletion_status
