# Supplementary material for: HIST1H2BJ is associated with poor prognosis and an immune-altered tumor microenvironment in glioma
Source: Front Immunol. 2026 Sep 16;17:1929885. doi: 10.3389/fimmu.2026.1929885 (PMC13624164; doi:10.3389/fimmu.2026.1929885)
Supplement: Supplementary file 4 [file Image5.pdf]

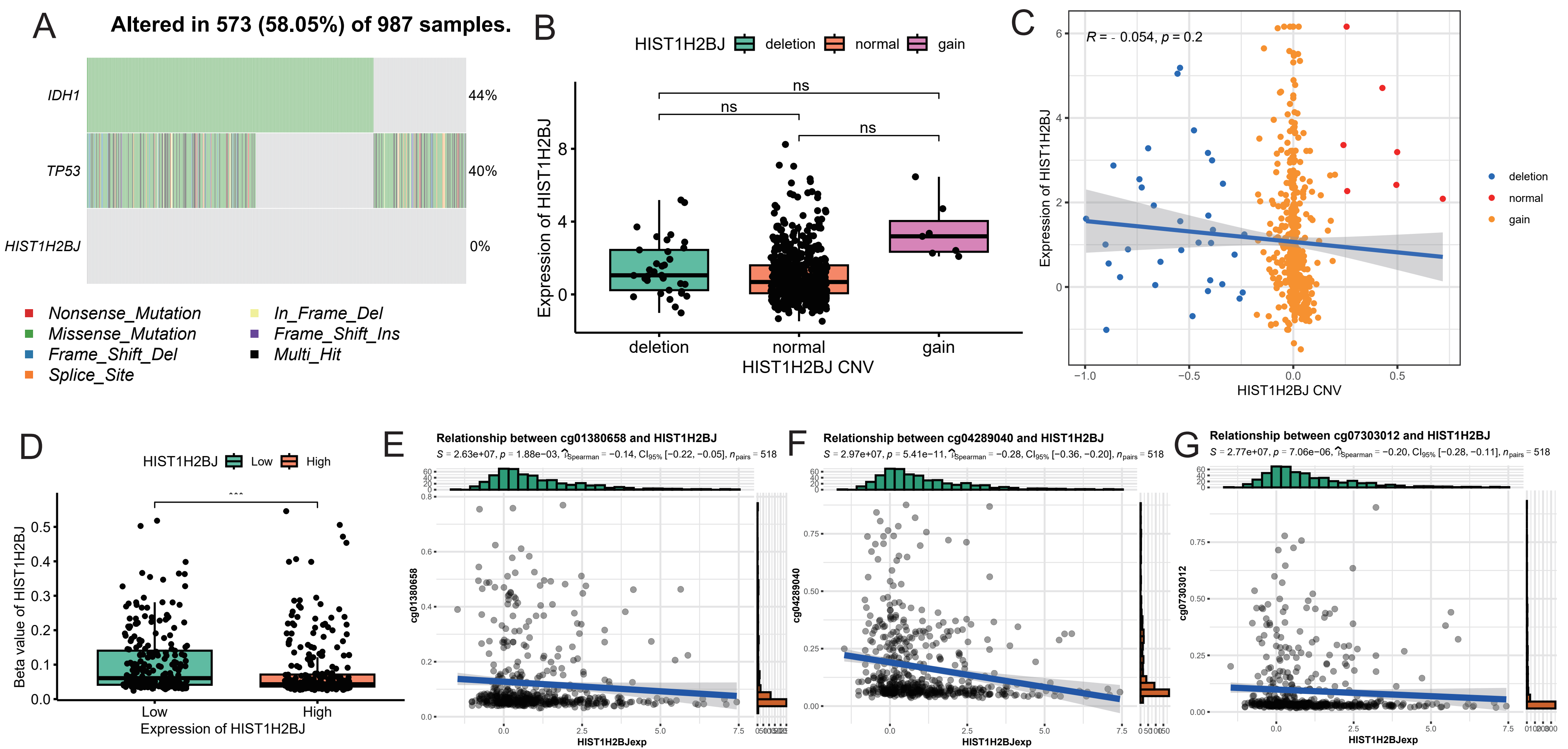

A
Altered in 573 (58.05%) of 987 samples.
IDH1 44%
TP53 40%
HIST1H2BJ 0%
Nonsense_Mutation
Missense_Mutation
Frame_Shift_Del
Splice_Site
In_Frame_Del
Frame_Shift_Ins
Multi_Hit
B
HIST1H2BJ deletion normal gain
ns
ns
ns
Expression of HIST1H2BJ
deletion normal gain
HIST1H2BJ CNV
C
R = - 0.054, p = 0.2
Expression of HIST1H2BJ
HIST1H2BJ CNV
deletion
normal
gain
D
HIST1H2BJ Low High
Beta value of HIST1H2BJ
Low High
Expression of HIST1H2BJ
E
Relationship between cg01380658 and HIST1H2BJ
S = 2.63e+07, p = 1.88e−03, ρ̂Spearman = −0.14, CI95% [−0.22, −0.05], npairs = 518
cg01380658
HIST1H2BJexp
F
Relationship between cg04289040 and HIST1H2BJ
S = 2.97e+07, p = 5.41e−11, ρ̂Spearman = −0.28, CI95% [−0.36, −0.20], npairs = 518
cg04289040
HIST1H2BJexp
G
Relationship between cg07303012 and HIST1H2BJ
S = 2.77e+07, p = 7.06e−06, ρ̂Spearman = −0.20, CI95% [−0.28, −0.11], npairs = 518
cg07303012
HIST1H2BJexp
