# Supplementary material for: HIST1H2BJ is associated with poor prognosis and an immune-altered tumor microenvironment in glioma
Source: Front Immunol. 2026 Sep 16;17:1929885. doi: 10.3389/fimmu.2026.1929885 (PMC13624164; doi:10.3389/fimmu.2026.1929885)
Supplement: Supplementary file 5 [file Image6.pdf]

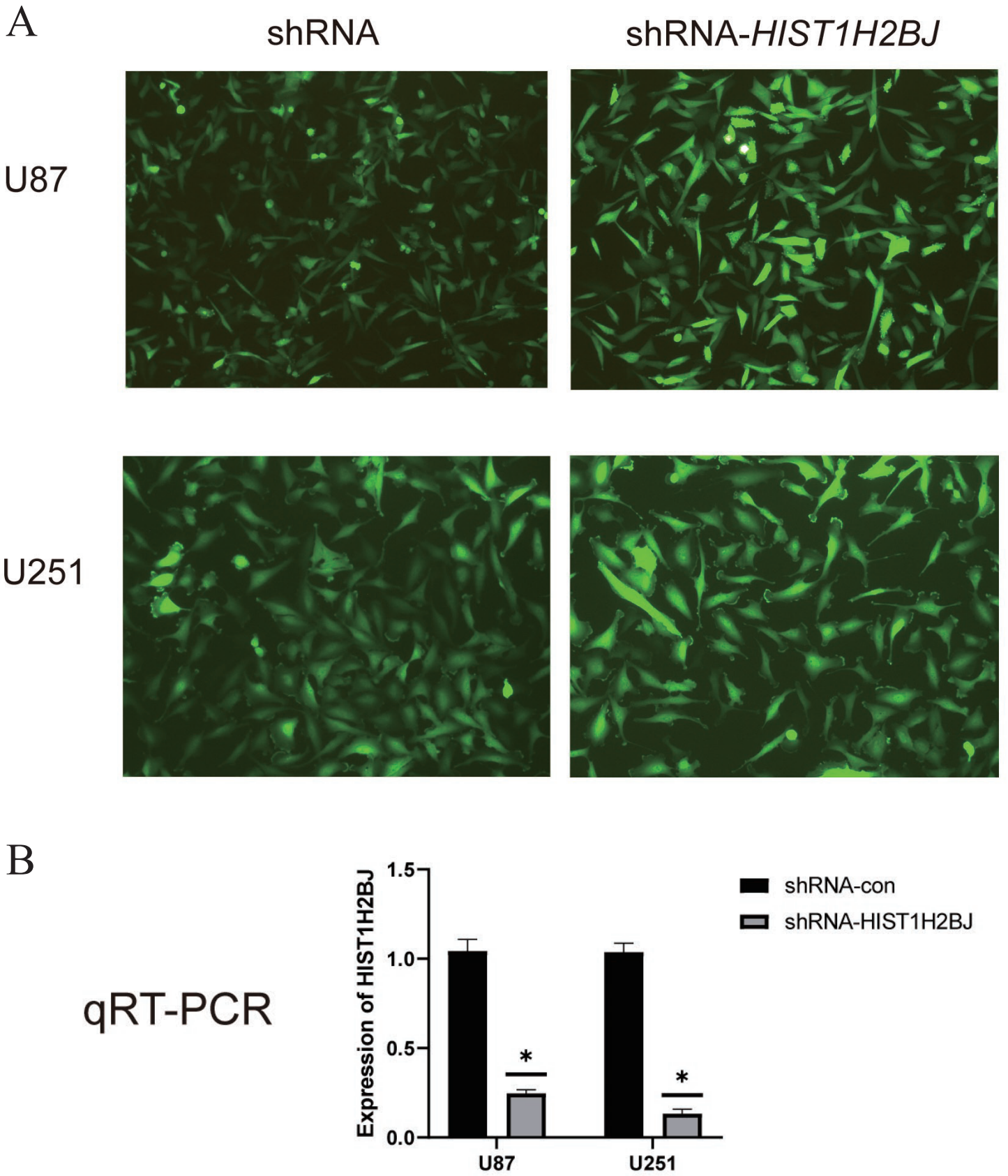
A
shRNA
shRNA-HIST1H2BJ
U87
U251
B
qRT-PCR
Expression of HIST1H2BJ
1.5
1.0
0.5
0.0
*
*
U87
U251
shRNA-con
shRNA-HIST1H2BJ
