# Supplementary material for: HIST1H2BJ is associated with poor prognosis and an immune-altered tumor microenvironment in glioma
Source: Front Immunol. 2026 Sep 16;17:1929885. doi: 10.3389/fimmu.2026.1929885 (PMC13624164; doi:10.3389/fimmu.2026.1929885)
Supplement: Supplementary file 6 [file Image7.pdf]

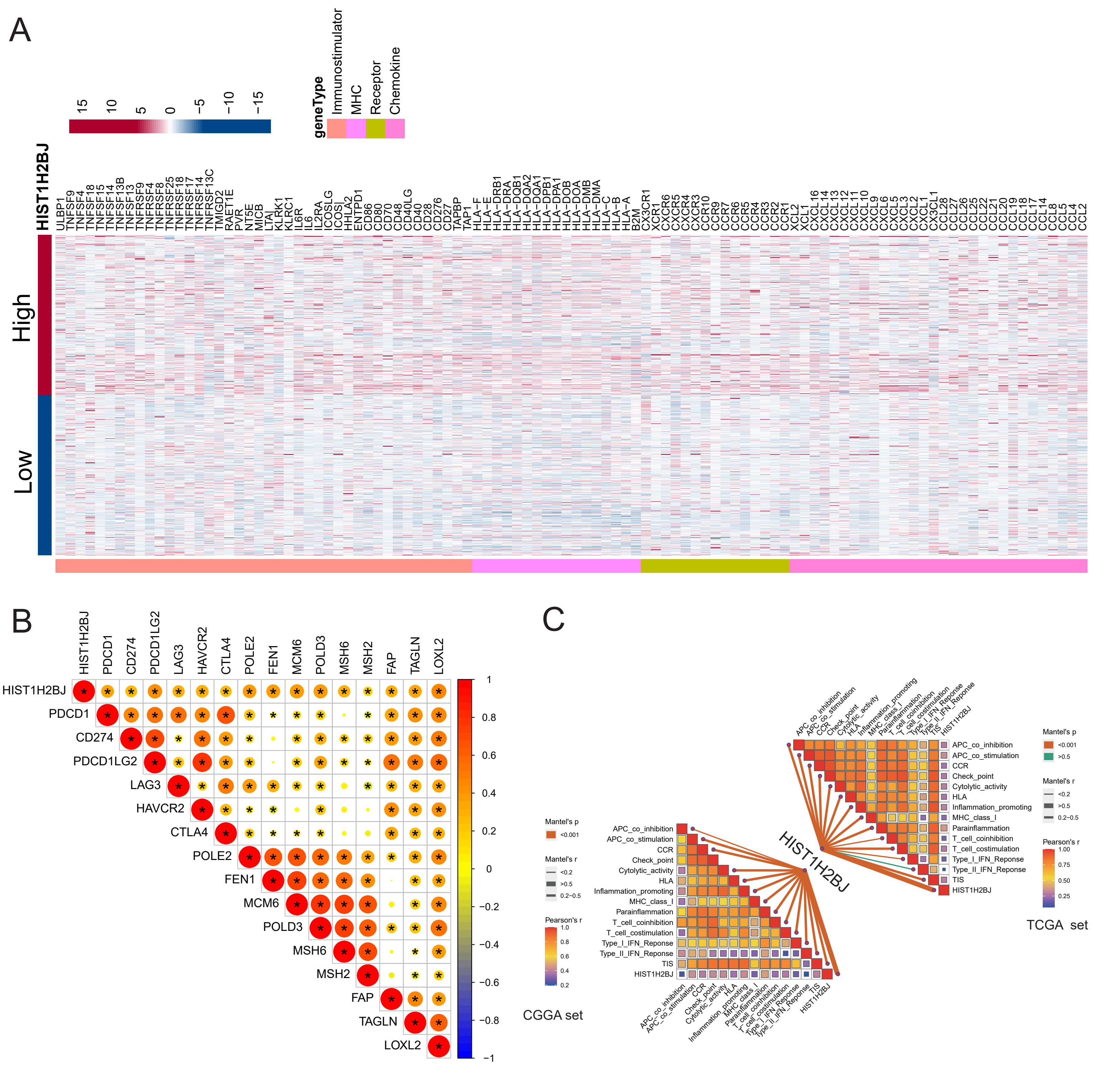

A
15 10 5 0 −5 −10 −15
geneType
Immunostimulator
MHC
Receptor
Chemokine
HIST1H2BJ
High
Low
ULBP1 TNFSF9 TNFSF4 TNFSF18 TNFSF15 TNFSF14 TNFSF13B TNFSF13 TNFRSF9 TNFRSF4 TNFRSF8 TNFRSF25 TNFRSF18 TNFRSF17 TNFRSF14 TNFRSF13C TMIGD2 RAET1E PVR NT5E MICB LTA KLRK1 KLRC1 IL6R IL6 IL2RA ICOSLG ICOS HHLA2 ENTPD1 CD86 CD80 CD70 CD48 CD40LG CD40 CD28 CD276 CD27 TAPBP TAP1 HLA−F HLA−E HLA−DRB1 HLA−DRA HLA−DQB1 HLA−DQA2 HLA−DQA1 HLA−DPB1 HLA−DPA1 HLA−DOB HLA−DOA HLA−DMB HLA−DMA HLA−C HLA−B HLA−A B2M CX3CR1 XCR1 CXCR6 CXCR5 CXCR4 CXCR3 CCR10 CCR9 CCR7 CCR6 CCR5 CCR4 CCR3 CCR2 CCR1 XCL2 XCL1 CXCL16 CXCL14 CXCL13 CXCL12 CXCL11 CXCL10 CXCL9 CXCL6 CXCL5 CXCL3 CXCL2 CXCL1 CX3CL1 CCL28 CCL27 CCL26 CCL25 CCL22 CCL21 CCL20 CCL19 CCL18 CCL17 CCL14 CCL8 CCL5 CCL4 CCL2
B
HIST1H2BJ PDCD1 CD274 PDCD1LG2 LAG3 HAVCR2 CTLA4 POLE2 FEN1 MCM6 POLD3 MSH6 MSH2 FAP TAGLN LOXL2
1 0.8 0.6 0.4 0.2 0 −0.2 −0.4 −0.6 −0.8 −1
C
HIST1H2BJ
APC_co_inhibition
APC_co_stimulation
CCR
Check_point
Cytolytic_activity
HLA
Inflammation_promoting
MHC_class_I
Parainflammation
T_cell_coinhibition
T_cell_costimulation
Type_I_IFN_Reponse
Type_II_IFN_Reponse
TIS
HIST1H2BJ
Mantel's p
<0.001
>0.5
Mantel's r
<0.2
>0.5
0.2−0.5
Pearson's r
1.0 0.8 0.6 0.4 0.2
1.00 0.75 0.50 0.25
CGGA set
TCGA set
