# Supplementary material for: HIST1H2BJ is associated with poor prognosis and an immune-altered tumor microenvironment in glioma
Source: Front Immunol. 2026 Sep 16;17:1929885. doi: 10.3389/fimmu.2026.1929885 (PMC13624164; doi:10.3389/fimmu.2026.1929885)
Supplement: Supplementary file 7 [file Image8.pdf]

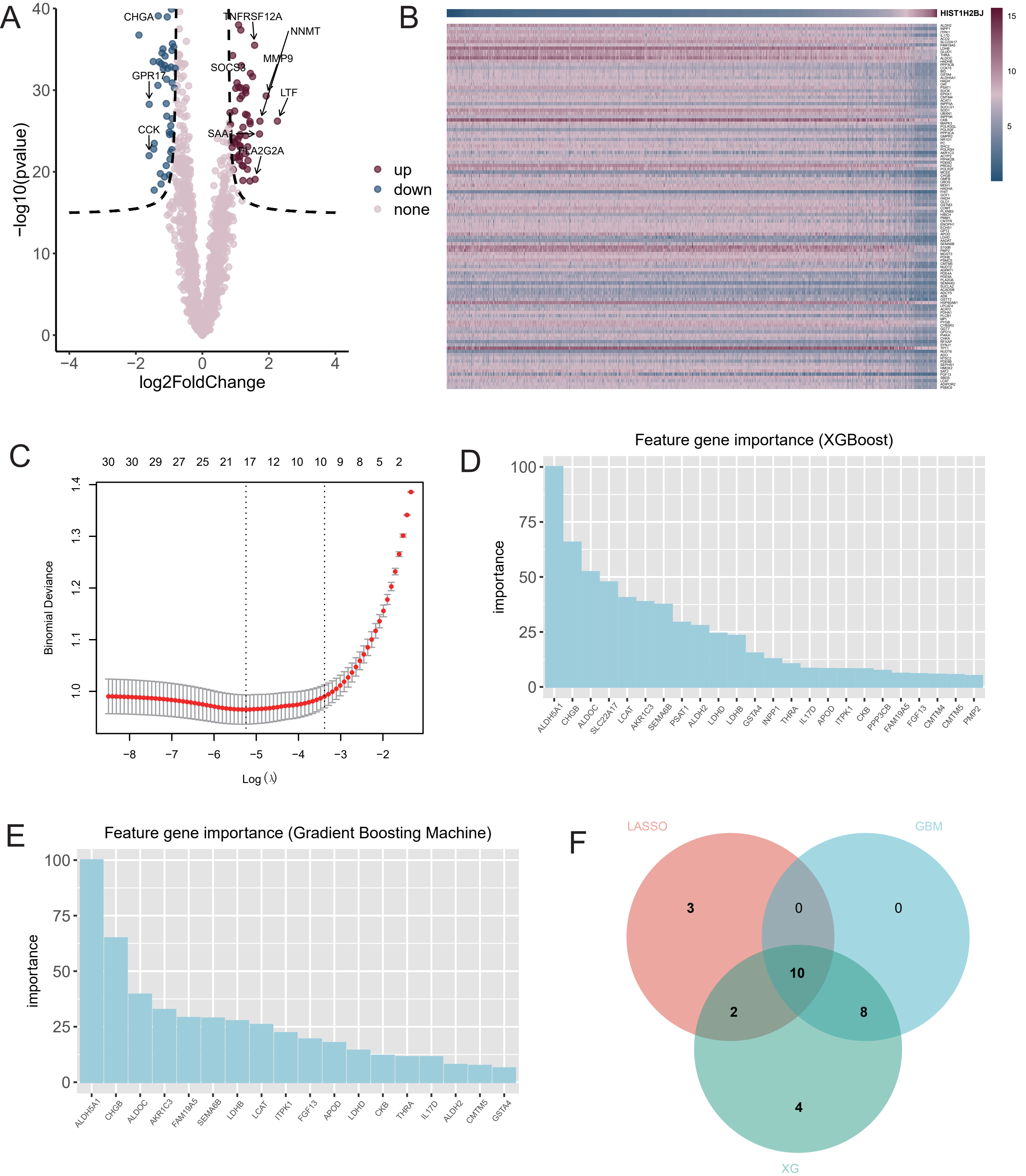

A
B
C
D
E
F
CHGA
TNFRSF12A
NNMT
MMP9
SOCS3
GPR17
LTF
CCK
SAA1
PLA2G2A
-log10(pvalue)
log2FoldChange
up
down
none
HIST1H2BJ
Binomial Deviance
Log (λ)
Feature gene importance (XGBoost)
importance
ALDH5A1
CHGB
ALDOC
SLC22A17
LCAT
AKR1C3
SEMA6B
PSAT1
ALDH2
LDHD
LDHB
GSTA4
INPP1
THRA
IL17D
APOD
ITPK1
CKB
PPP3CB
FAM19A5
FGF13
CMTM4
CMTM5
PMP2
Feature gene importance (Gradient Boosting Machine)
importance
ALDH5A1
CHGB
ALDOC
AKR1C3
FAM19A5
SEMA6B
LDHB
LCAT
ITPK1
FGF13
APOD
LDHD
CKB
THRA
IL17D
ALDH2
CMTM5
GSTA4
LASSO
GBM
XG
3
0
0
10
2
8
4
