# Supplementary material for: HIST1H2BJ is associated with poor prognosis and an immune-altered tumor microenvironment in glioma
Source: Front Immunol. 2026 Sep 16;17:1929885. doi: 10.3389/fimmu.2026.1929885 (PMC13624164; doi:10.3389/fimmu.2026.1929885)
Supplement: Supplementary file 8 [file Image9.pdf]

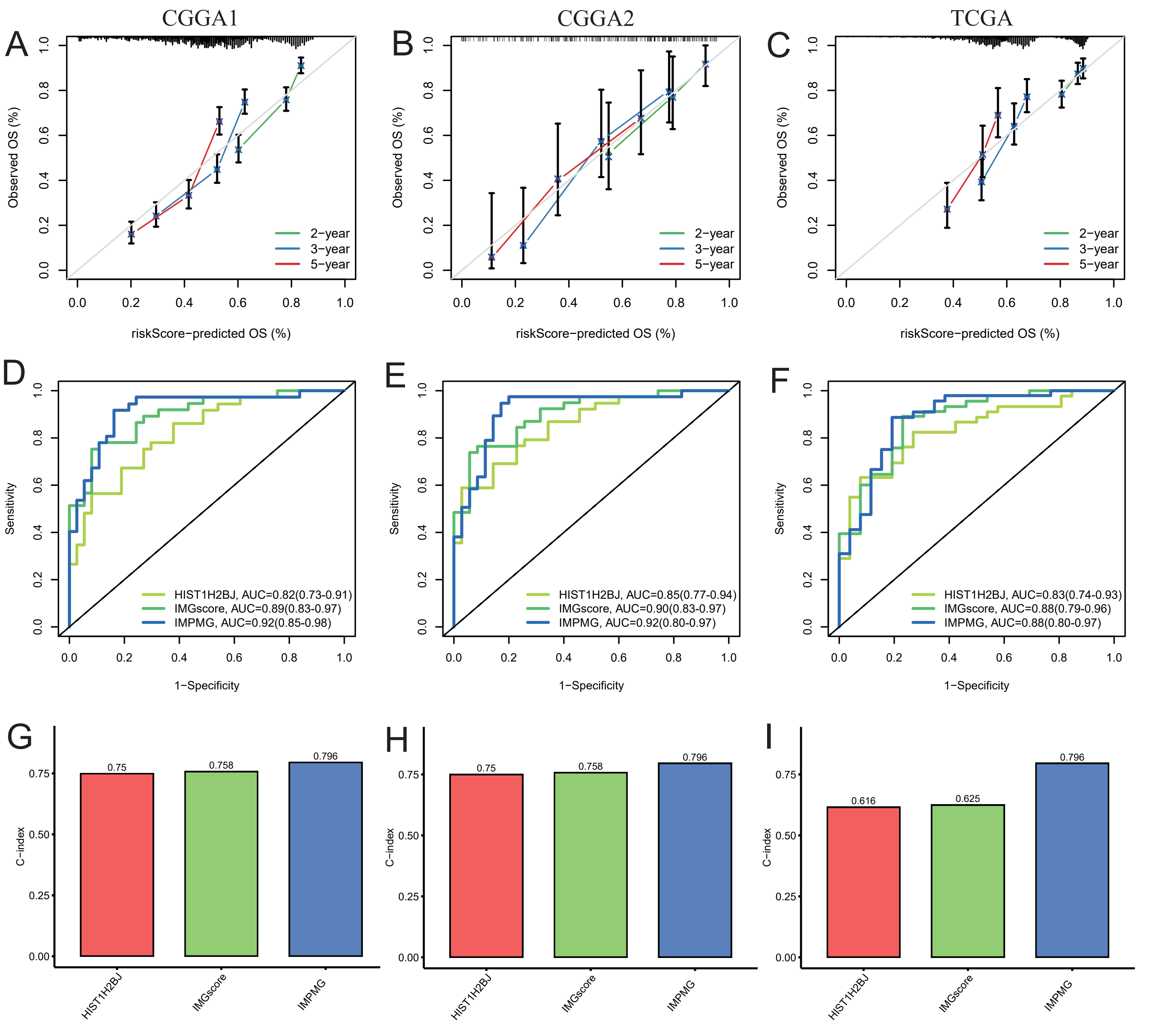

A
CGGA1
B
CGGA2
C
TCGA
Observed OS (%)
riskScore−predicted OS (%)
2−year
3−year
5−year
D
E
F
Sensitivity
1−Specificity
HIST1H2BJ, AUC=0.82(0.73-0.91)
IMGscore, AUC=0.89(0.83-0.97)
IMPMG, AUC=0.92(0.85-0.98)
HIST1H2BJ, AUC=0.85(0.77-0.94)
IMGscore, AUC=0.90(0.83-0.97)
IMPMG, AUC=0.92(0.80-0.97)
HIST1H2BJ, AUC=0.83(0.74-0.93)
IMGscore, AUC=0.88(0.79-0.96)
IMPMG, AUC=0.88(0.80-0.97)
G
H
I
C−index
0.75
0.758
0.796
0.75
0.758
0.796
0.616
0.625
0.796
HIST1H2BJ
IMGscore
IMPMG
