# Supplementary material for: HIST1H2BJ is associated with poor prognosis and an immune-altered tumor microenvironment in glioma
Source: Front Immunol. 2026 Sep 16;17:1929885. doi: 10.3389/fimmu.2026.1929885 (PMC13624164; doi:10.3389/fimmu.2026.1929885)
Supplement: Supplementary file 9 [file DataSheet3.docx]

HIST1H2BJ Is Associated with Poor Prognosis and an Immune-Altered Tumor Microenvironment in Glioma

Lupeng Zhang^1#^, Yue Li^2#^, Chen Shi^1#^, Yong Zhang^3^, Ruijuan Liu^6^, Chiwen Qu^4*^, Sihua Peng^5*^, Xiaoning Peng^1,3*^

Supplementary Material

**1 Supplementary methods**

**Acquisition and preprocessing of glioma data from public databases**

Gene expression data and clinical information on glioma were obtained from the CGGA and TCGA databases. The CGGA database included two independent cohorts: CGGA1, comprising RNA sequencing data (dataset IDs: mRNAseq_693 and mRNAseq_325), and CGGA2, comprising microarray data (dataset ID: mRNA-array_301). The data of glioma somatic mutation, copy number variation (data type: Masked Copy Number Segment hg38), and DNA methylation (data type: Illumina Human Methylation 450) were obtained from the TCGA database. We excluded patients with incomplete survival information and clinical information. To eliminate the variation caused by different sample preservation methods, we excluded formalin-fixed paraffin-embedded tumor samples in the TCGA cohort. Moreover, we transformed the RNA-sequencing data (FPKM values) into transcripts per kilobase million (TPM) format. Batch effect correction was performed on the two RNA sequencing datasets from the CGGA1 cohort (mRNAseq_693 and mRNAseq_325) using the 'SVA' package.

**Identification of crucial prognostic gene for glioma**

To completely eliminate information leakage and identify robust prognostic biomarkers, we established a rigorous Bootstrap resampling framework (B = 500) using glioma transcriptome data from the CGGA1 dataset. In each iteration, a Bootstrap training set was constructed via random sampling with replacement. Within each Bootstrap training set, the analyses were conducted as follows:

(1) Kaplan–Meier survival analysis combined with univariate Cox regression analysis was performed to identify genes significantly associated with overall survival (OS) in glioma patients. Prior to screening, genes with an expression standard deviation (SD) less than 0.1 across samples were excluded. For each gene, patients were dichotomized into high- and low-expression groups according to the median expression level, and the survival difference between the two groups was evaluated by the log-rank test. Genes meeting both a significance threshold of P < 0.001 and an absolute difference in the 5-year survival rate greater than 0.2 between the high- and low-expression groups were retained, and the hazard ratio (HR) and 95% confidence interval (CI) of each gene were recorded. Only genes with a selection frequency > 70% across all iterations were carried forward.

(2) For each candidate gene identified in the first step, a separate multivariate Cox proportional hazards model was constructed. In each model, the expression of the candidate gene was analyzed together with prespecified clinical covariates, including age, sex, WHO grade, IDH mutation status, 1p/19q co-deletion status, and PRS type. After adjusting for these clinical covariates, genes that remained significantly associated with OS (P < 0.001) were defined as "independent prognostic genes", and the corresponding HRs and 95% CIs were recorded. Only genes with a selection frequency > 70% were carried forward.

(3) The genes screened in the prior step were evaluated by receiver operating characteristic (ROC) curve analysis using the survivalROC R package, with 1-year survival as the evaluation time point (method = "KM"), to calculate the area under the curve (AUC). Genes with AUC > 0.7 were retained. Only genes with a selection frequency > 70% were carried forward.

(4) The association between the retained genes and clinical features was examined using the Wilcoxon rank-sum test for binary clinical variables and the Kruskal–Wallis test for variables with three or more categories (P < 0.05). Genes significantly associated with at least seven clinical features were retained. Only genes with a selection frequency > 70% were carried forward.

For the significance-based screening procedures described above, P values were corrected for multiple testing using the Benjamini–Hochberg method, and all retained genes satisfied a false discovery rate (FDR) < 0.05.

The genes retained from the preceding four-step screening pipeline were then independently subjected to each of the following four machine-learning methods. For each algorithm, a separate Bootstrap resampling procedure with 500 iterations was performed, and only genes with a selection frequency > 70% across all iterations were retained as the core gene set for that algorithm:

(1) XGBoost, implemented through the caret R package (method = "xgbTree"), with hyperparameters automatically tuned by grid search and model performance evaluated by 10-fold cross-validation repeated 4 times (random seed = 123); genes with an importance score greater than 5 were kept.

(2) The gradient boosting machine (GBM), implemented through the caret R package (method = "gbm"), with hyperparameters tuned by grid search and performance evaluated by 4-fold cross-validation repeated 4 times (random seed = 1234); genes with an importance score greater than 5 were kept.

(3) LASSO regression, implemented using the glmnet R package with a binomial logistic model (alpha = 1); ten-fold cross-validation with binomial deviance as the loss function was performed, and genes with non-zero coefficients at lambda.min were retained to eliminate redundant genes.

(4) Random forest, implemented using the randomForest R package, with the number of trees set to 500 (ntree = 500). Gene importance was measured by the mean decrease in the Gini index (MeanDecreaseGini), and genes with an importance score greater than 5 were kept.

Finally, to obtain the key genes affecting the prognosis of glioma patients, we calculated the intersection of the four core gene sets obtained from the above machine-learning algorithms and the differentially expressed genes of CD8+ T cells.

**Analysis of the prognostic value of HIST1H2BJ in glioma**

Correlations between *HIST1H2BJ* and different clinical characteristics (Age, RPS type, Histology, Grade, Gender, *IDH* status, 1p/19q co-deletion status, Chemo_status, Radio_status) were analyzed using Kruskal-Wallis and Mann-Whitney tests. Multivariate Cox analysis was used to determine whether *HIST1H2BJ* is an independent prognostic factor for glioma patients.

**Functional Enrichment Analysis**

Gene set enrichment analysis is a computational method used to determine whether a group of genes is differentially expressed in two biological states. In this study, GSEA was used to generate an ordered list of all genes associated with the expression of *HIST1H2BJ*. Then, GSEA was used to identify survival differences between the high and low *HIST1H2BJ* groups. Gene set permutations were performed one thousand times for each analysis. The expression level of *HIST1H2BJ* was used as a phenotype label. The false discovery rate (FDR) and normalized enrichment score (NES) were used to sort the gene ontology (GO) and KEGG pathways enriched in each phenotype.

**Somatic mutation analysis**

Somatic mutation information of glioma was retrieved from the TCGA database. The data which included somatic variants were reserved in the Mutation Annotation Format (MAF) form. The "maftool" package was used to identify high-frequency mutant genes (mutation frequency ≥ 5%) in *HIST1H2BJ* high expression and *HIST1H2BJ* low expression groups patients, and then the difference of high-frequency mutant genes between *HIST1H2BJ* high expression and *HIST1H2BJ* low expression groups was determined by chi-square test (*P* < 0.05, FDR < 0.05).

**Somatic copy number variation analysis**

The Gistic 2.0 module of GenePattern 2.0 was used to identify significant SCNAs in *HIST1H2BJ* high expression and *HIST1H2BJ* low expression groups in glioma patients. The parameters are as follows: -ta, 0.2; -td, 0.2; -js, 100; -brlen, 0.99; -conf, 0.95; -remove x 1; and -q-value, 0.05. Then, the chi-square test was used to determine the difference in copy number variation rate of genes carried in the overlapping region between *HIST1H2BJ* high expression and *HIST1H2BJ* low expression groups (*P* < 0.05, FDR < 0.05). The Circos diagram is drawn by the R package " Rciorcos ".

**Immunohistochemical (IHC) staining**

After deparaffinization and dehydration, glioma tissues and non-tumor brain tissue slices were incubated with polyclonal anti-*HIST1H2BJ* antibody (dilution 1:300, Abcepta AP10727c, China) for 90 minutes. Then, slices were incubated with an anti-rabbit secondary antibody (SM802, DOKA, Denmark) for 30 min, and the signal was detected with Diaminobenzidine (DAB, Thermo Fisher Scientific, USA). Staining was observed separately under the microscope by two pathologists. It was judged as positive if the nuclear staining showed yellow or brown. The expression level of *HIST1H2BJ* was determined by calculating the positive cell rate and positive degree.

**Establishment of *HIST1H2BJ*-knockdown stable cell lines**

Lentiviral shRNA constructs targeting the human HIST1H2BJ (official gene symbol H2BC11, NM_021058) were designed and synthesized by GeneChem (Shanghai, China). Three independent shRNA sequences were designed via the Easy-siRNA algorithm to target the coding sequence (CDS, nt 46–426) of *HIST1H2BJ* : shRNA#1 (clone 106264-1), 5'-atTCGTTTGTGAACGACATTT-3'; shRNA#2 (clone 106265-1), 5'-GGGCATCATGAATTCGTTTGT-3'; and shRNA#3 (clone 106266-2), 5'-GCAAACTCTCAACCCTAACGG-3'. The synthesized shRNA oligonucleotides were inserted into the GV493 lentiviral vector (hU6-MCS-CBh-gcGFP-IRES-puromycin; GeneChem), and allrecombinant lentiviruses were verified by DNA sequencing.

A scrambled non-targeting shRNA (CON313, 5'-TTCTCCGAACGTGTCACGT-3') served as the negative control (NC). The results of the recombination lentivirus titration are as follows: shRNA#1 has a titer of 1.0 × 10⁹ TU/mL, while shRNA#2 and shRNA#3 both have a titer of 8.0 × 10⁸ TU/mL.. Cell line were seeded into 6-well plates at approximately 20% confluency and infected with lentivirus in the presence of HiTransG A transduction enhancer (GeneChem) at a multiplicity of infection (MOI) of 5 for U87 and 10 for U251. The negative control virus (CON313, 5.0 × 10⁸ TU/mL) was used in parallel. After 72 h, transduction efficiency was monitored by green fluorescent protein (GFP) expression under a fluorescence microscope. Stable cell lines were subsequently selected with puromycin for approximately 14 days. The knockdown efficiency of *HIST1H2BJ* was validated by quantitative real-time PCR (qRT-PCR) using primers specifically targeting a unique sequence of HIST1H2BJ distinct from other H2B family members; melting curve analysis confirmed a single sharp peak, indicating specific amplification without primer dimers. Based on the qRT-PCR results, shRNA#2 (clone 106265-1), which achieved efficient *HIST1H2BJ* knockdown, was selected for use in subsequent functional assays.

**RNA extraction and qRT-PCR**

Total RNA was extracted from GBM cell lines U87 and U251 using Trizol reagent (Invitrogen, USA), and the RNA concentration was detected by spectrophotometry. RNA samples were reverse transcribed into cDNA according to the instructions of the M-MLV Reverse Transcriptase (Promega, USA), and the HIST1H2BJ mRNA level was analyzed by SYBR green PCR kit (Vazyme Biotech, Nanjing, China) in the CFX96 real-time PCR system (Bio-Rad, USA). The relative expression level of HIST1H2BJ mRNA was calculated by the 2-△△CT method, and β-actin was used as an internal reference. All experiments were performed in triplicate. The gene primer sequences are as follows:

| Gene | Forward primer（5'-3'） | Reverse primer（5'-3'） |
| --- | --- | --- |
| HIST1H2BJ | 5' - TGTTTTTCCTTTTCGTTGGCG-3' | 5'- GTGGACCTGCTTCAGAACCTTG-3' |
| β-actin | 5'- GCGTGACATTAAGGAGAAGC-3' | 5'-CCACGTCACACTTCATGATGG-3' |

**2 Supplementary Result**

**Development and construction of the IMPMG model**

Our previous analysis discovered that *HIST1H2BJ* predominantly influences immune and metabolism-related signaling pathways in glioma. We conducted differential expression gene (DEG) analysis on immune and metabolic genes between *HIST1H2BJ*^high^ and *HIST1H1BJ*^low^ and identified 189 DEGs (Figure S8A). Then, correlation analysis found 113 co-expressed genes of *HIST1H2BJ* (Figure S8B). The intersection of DEGs and co-expressed genes yielded 31 hub genes. Using the LASSO regression, XGboost, and Gradient Boosting Machine algorithms to analyze these 31 genes, we obtained 15, 24, and 18 genes, respectively. Further, taking the intersection of the gene identified by the above algorithm, we obtained ten core genes related to *HISTH2BJ* (Figure S8C-F). In the CGGA1 cohort, we constructed the IMGscore by performing stepwise multivariate Cox regression on these ten signature genes. Finally, we integrated *HIST1H2BJ*, IMGscore, and clinical prognostic factors to build the IMPMG model using stepwise multivariate Cox regression analysis, and it was defined as:

IMPMG_score=0.2104×*HIST1H2BJ*+0.7126×PRS_type+0.5562×grade+0.2803×age-0.2632×*IDH*-0.7832×1p/19q+0.2017×IMGscore

IMGscore=0.2346×*THRA*+0.1667×*GSTA4*-0.4670×*ALDH5A1*-0.1733×*CHGB*-0.1047×*ALDOC*-0.1671×*ITPK1*-0.1584×*AKR1C3*-0.1210×*SEMA6B*-0.1876×*LCAT*

**3 Supplementary Figures**

Figure S1. Screening of hub genes via machine learning. Venn diagram illustrating the intersecting genes among Random Forest (RF), LASSO, Gradient Boosting Machine (GBM), XGBoost, and differentially expressed genes (DEGs) of CD8+ T cells.

Figure S2. Analysis of HIST1H2BJ Expression based on GEPIA and HPA databases.

(A) differential expression of *HIST1H2BJ* between non-tumor tissues and LGG in the GEPIA database; (B) differential expression of HIST1H2BJ between non-tumor tissues and GBM in the GEPIA database; (C-E) non-tumor tissues detected by immunohistochemical results in HPA database (C) Protein levels of HIST1H2BJ in LGG (D) and GBM tissues(E).

Figure S3. Correlation analysis between HIST1H2BJ expression and clinical characteristics using the CGGA database.

(A-F) The expression of *HIST1H2BJ* was correlated with (A) RPS type (Primary, N = 500; Recurrent, N = 243; Secondary, N = 25), (B) grade (WHO Ⅱ, N = 222; WHO Ⅲ, N = 251; WHO Ⅳ, N = 303), (C) Age (≤ 41, N = 355; > 41, N = 421), (D) Chemo status (No, N = 240; Yes, N = 536), (E) IDH mutation status (Mutant, N = 427; Wildtype, N = 349), (F) 1p/19q codeletion status (Codel, N = 164; Non-codel, N = 612).

Figure S4. Analysis of HIST1H2BJ Expression in Glioma Cells and Clinical Samples.

(A) The mRNA levels of *HIST1H2BJ* in glioma cell lines and normal glial cells were measured by qRT-PCR. (B) IHC staining images of HIST1H2BJ in non-tumor and glioma tissue.

Figure S5. Analysis of mutations, CNV, and DNA methylation of the HIST1H2BJ gene in glioma patients.

(A) The mutational landscape of *HIST1H2BJ* in glioma. (B-C) Correlation analysis between CNV and expression of HIST1H2BJ in glioma patients. (D-G) correlation between the expression of *HIST1H2BJ* and the methylation levels of cg01380658, cg04289040, and cg07303012 probes in glioma patients.

Figure S6. Construction of *HIST1H2BJ* knockdown stable glioma cell line.

The efficacy of infection of lentiviruses expressing shRNA-con and shRNA-HIST1H2BJ to with U87, U251 was detected by fluorescence microscopy and qRT-PCR.

Figure S7. Correlation analysis between HIST1H2BJ and TME in glioma patients.

(A) Differences in the expression of 122 immunomodulators (chemokines, receptors, MHC, and immunostimulators) between *HIST1H2BJ*^low^ and *HIST1H2BJ*^high^ in glioma. (B) Correlation between *HIST1H2BJ* and 15 inhibitory immune checkpoints. (C) Correlation between *HIST1H2BJ* and immune indicators.

Figure S8. Using machine learning to screen immune and metabolic function-related genes associated with HIST1H2BJ.

(A) Differential analysis of immune and metabolic-related genes between the *HIST1H2BJ*^high^ and the *HIST1H2BJ*^low^. (B) Correlation analysis between *HIST1H2BJ* expression and immune and metabolic genes. (C-E) Feature gene selection using LASSO regression analysis (C), XGboost (D), and GBM algorithm (E). (F) Intersection genes are identified by the three algorithms.

Figure S9. Accuracy of IMPMG in predicting the prognosis of glioma patients. (A-C) Calibration curves for IMPMG predicting 2-year, 3-year, and 5-year survival in glioma patients were plotted in the CGGA1 (A), CGGA2 (B), and TCGA (C) datasets, respectively. (D-F) ROC curves were plotted for IMPMG, *HIST1H2BJ*, and IMG_score in the CGGA1 dataset, comparing differences in AUC values. (G-I) Comparison of the C-index for IMPMG, *HIST1H2BJ*, and IMG_score in the CGGA1 dataset
